# Supplementary material for: Serious bacterial infections and antibiotic prescribing in primary care: cohort study using electronic health records in the UK
Source: BMJ Open. 2020 Feb 28;10(2):e036975. doi: 10.1136/bmjopen-2020-036975 (PMC7050314; doi:10.1136/bmjopen-2020-036975)
Supplement: Supplementary data [file bmjopen-2020-036975supp001.pdf]

Gulliford et al. Supplementary Data

Supplementary Table 1: Outline of statistical models employed for analysis. (Please see text for further explanation).

**Model 1**	Antibiotic_count ~ year+year^2+year^3+age_group+gender+comorbidity+deprivation+region+offset=log(person-years)+random_intercept(family_practice), model=Poisson
**Model 2**	Serious_bacterial_infection_count ~ Family_Practice_AB_Prescribing_Estimate+Family_Practice_Consultations_Coded_Estimate+year+year^2+year^3+gender+ age_group+deprivation+region+offset=log(person-years)+random_intercept(family_practice), model=Poisson

Gulliford MC, *et al. BMJ Open* 2020; 10:e036975. doi: 10.1136/bmjopen-2020-036975

Gulliford et al. Supplementary Data

Figure 1: Flowchart showing classification of antibiotic prescriptions 2002 to 2017. Figures are frequencies (percent of total number of antibiotic prescriptions).

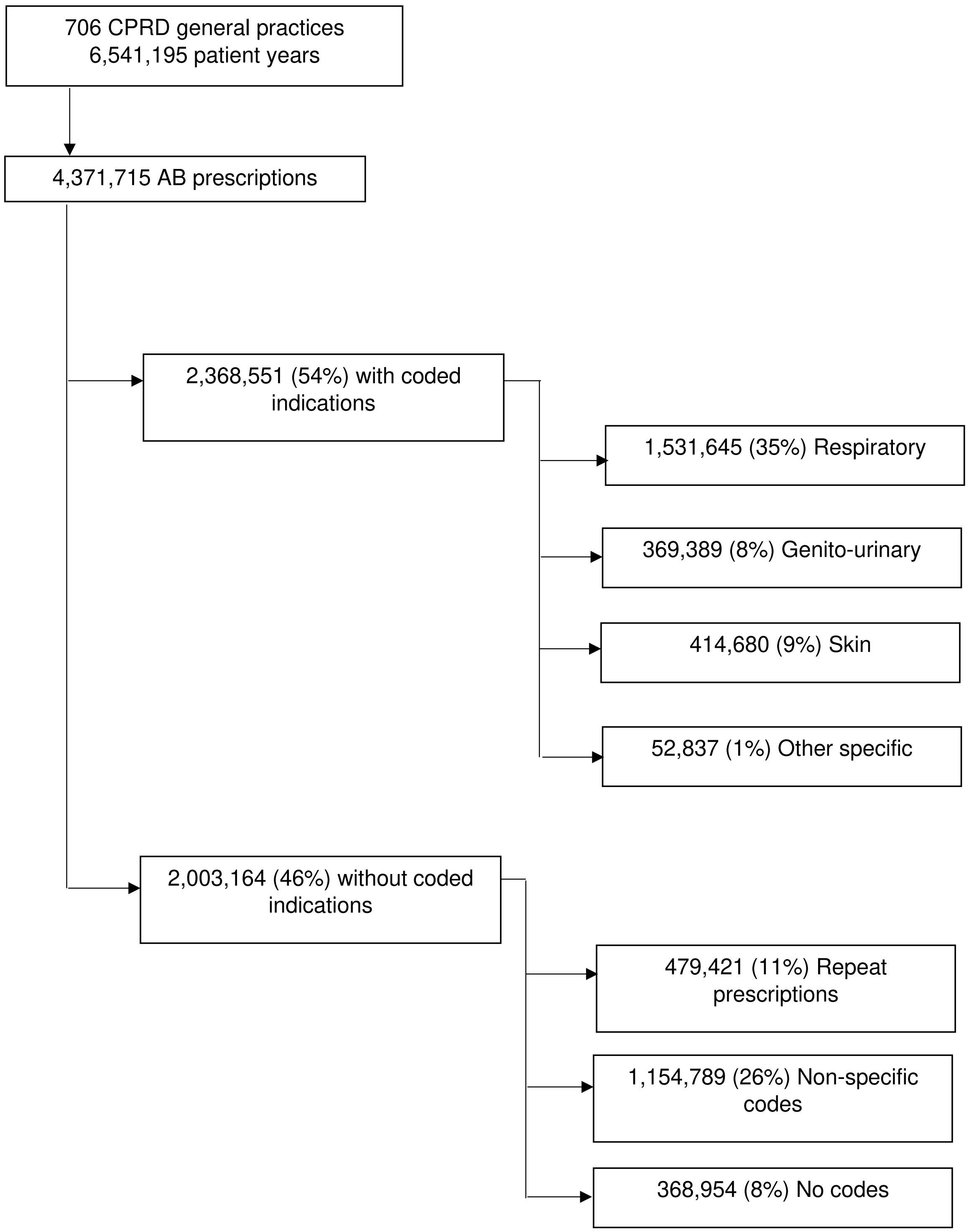

Gulliford MC, *et al. BMJ Open* 2020; 10:e036975. doi: 10.1136/bmjopen-2020-036975

Gulliford et al. Supplementary Data

Supplementary Table 2: Characteristics of sample and antibiotic prescriptions by time period. Figures are frequencies (column percent).

	2002 to 2006	2007 to 2012	2013 to 2017
**Number of general practices**	652	672	589
**Number of patients contributing person time**[a]	548,558	576,985	439,627
**Number of person years**	2,253,436	2,768,176	1,519,582
Age 0 to 4	275,539	313,806	104,688
Age 5 to 14	371,352	611,610	393,224
Age 85+	169,709	216,966	111,606
Comorbidity present[a]	835,565	1,147,828	686,777
**Number of antibiotic prescriptions**	1,422,009	1,941,102	1,008,604
Acute AB prescriptions	1,289,615 (91)	1,739,666 (90)	863,013 (86)
for RTI	534,535 (38)	705,262 (36)	291,848 (29)
for GUTI	115,928 (8)	166,336 (9)	87,125 (9)
for skin infection	137,936 (10)	184,420 (10)	92,324 (9)
Other specific codes recorded	18,277 (1)	24,849 (1)	9,711 (1)
Non-specific codes recorded	290,472 (20)	537,110 (28)	327,207 (32)
No codes recorded	192,467 (14)	121,689 (6)	54,798 (5)
Repeat AB prescriptions	132,394 (9)	201,436 (10)	145,591 (15)

[a] figures were rounded to nearest whole number; [b]based on seasonal-influenza risk status

Gulliford MC, *et al. BMJ Open* 2020; 10:e036975. doi: 10.1136/bmjopen-2020-036975

Gulliford et al. Supplementary Data

Supplementary Figure 2: Age-standardised antibiotic prescribing rates per 1,000 patient years for males and females from 2002 to 2017.

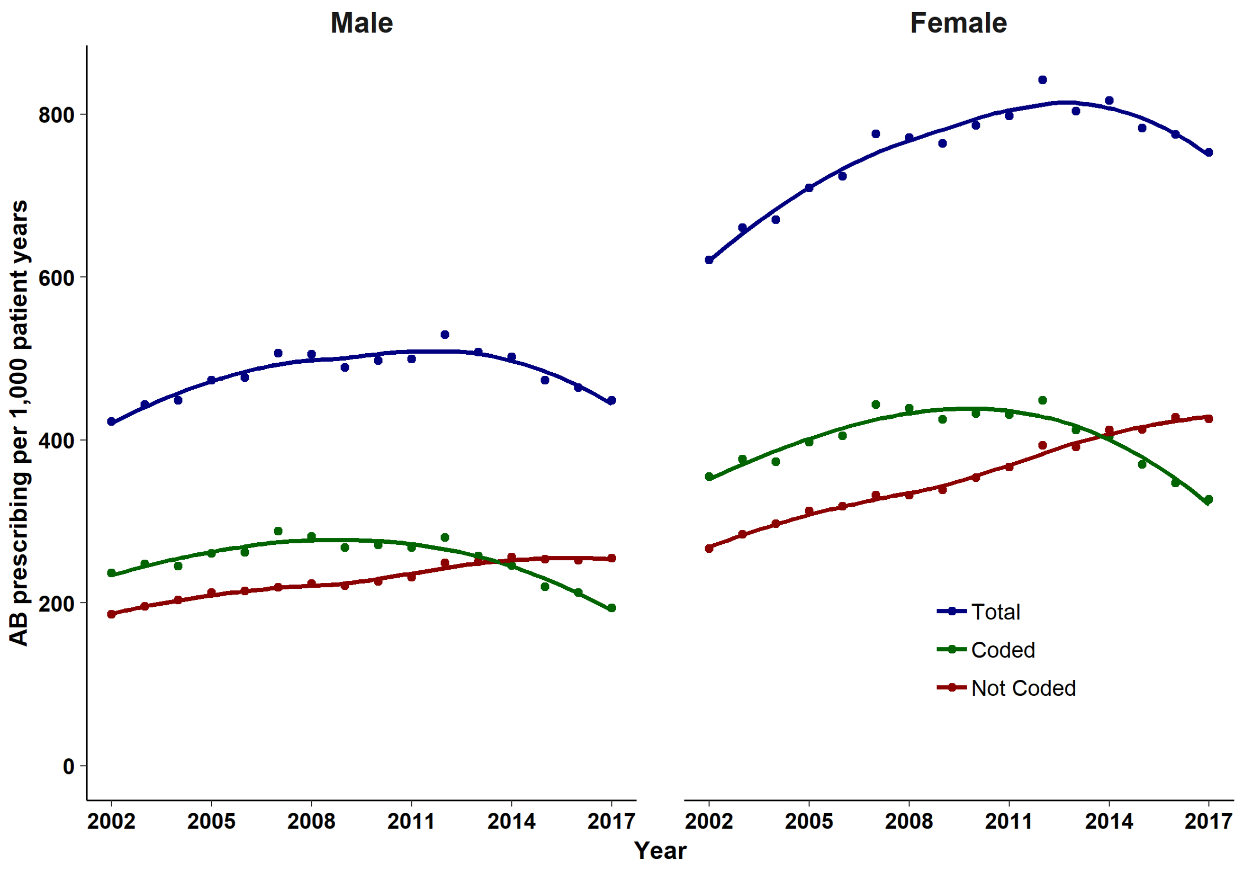

Gulliford MC, *et al. BMJ Open* 2020; 10:e036975. doi: 10.1136/bmjopen-2020-036975

Gulliford et al. Supplementary Data

Supplementary Table 3: Distribution of serious bacterial infection events 2002 to 2017 by age-group and gender. Figures are frequencies.

	CNS infection	CVS infection	Kidney infection	Lung abscess / empyema	Mastoiditis	Osteomyelitis	Peritonsillar abscess	AB resistant infections	Sepsis	Septic arthritis
All	576	1,697	30,827	2,932	1,970	4,921	11,338	42,185	39,059	4,254
Male	352	1,144	4,997	1,903	814	3,055	6,021	18,312	18,999	2,496
Female	224	553	25,830	1,029	1,156	1,866	5,317	23,873	20,060	1,758
**Age-group (years)**										
0 to 4	11	20	198	138	178	138	73	576	469	147
5 to 9	17	18	386	106	153	118	232	409	334	104
10 to 14	17	17	474	60	111	167	465	308	244	93
15 to 24	47	42	6,140	106	167	152	3,428	1,528	970	129
25 to 34	38	92	5,523	149	203	160	2,621	2,444	1,474	243
35 to 44	65	146	5,176	294	280	392	2,483	3,089	2,164	392
45 to 54	115	189	4,519	438	270	635	1,079	4,001	3,345	555
55 to 64	105	274	3,725	561	255	865	553	5,045	5,385	678
65 to 74	90	407	2,562	525	210	937	285	8,252	7,817	775
75 to 84	58	365	1,548	423	109	924	94	9,469	9,646	727
85+	13	127	576	132	34	433	24	7,064	7,211	411

Gulliford MC, *et al. BMJ Open* 2020; 10:e036975. doi: 10.1136/bmjopen-2020-036975

Gulliford et al. Supplementary Data

Supplementary Table 4: Age- and sex-standardised incidence rates per 1,000 patient-years for serious bacterial infections by quartile of antibiotic prescribing.

	General practice antibiotic prescribing[a]		General practice medical coding[a]	
**Group**	**Lowest fourth of general practices**	**Highest fourth of general practices**	**Lowest fourth of general practices**	**Highest fourth of general practices**
All	1.86 (1.83 to 1.88)	2.23 (2.20 to 2.25)	1.94 (1.91 to 1.96)	2.39 (2.37 to 2.42)
CNS Infection	0.008 (0.007 to 0.010)	0.009 (0.008 to 0.011)	0.008 (0.007 to 0.009)	0.010 (0.009 to 0.012)
CVS infection	0.024 (0.021 to 0.027)	0.026 (0.023 to 0.028)	0.026 (0.024 to 0.029)	0.027 (0.025 to 0.030)
Kidney Infection	0.40 (0.39 to 0.41)	0.49 (0.48 to 0.50)	0.37 (0.37 to 0.38)	0.55 (0.53 to 0.56)
Lung abscess / empyema	0.042 (0.039 to 0.045)	0.045 (0.042 to 0.049)	0.044 (0.041 to 0.047)	0.049 (0.046 to 0.053)
Mastoiditis	0.025 (0.022 to 0.027)	0.033 (0.030 to 0.036)	0.021 (0.019 to 0.023)	0.036 (0.033 to 0.039)
Osteomyelitis	0.071 (0.067 to 0.075)	0.073 (0.069 to 0.077)	0.071 (0.067 to 0.075)	0.081 (0.077 to 0.086)
Peritonsillar abscess	0.16 (0.15 to 0.17)	0.16 (0.16 to 0.17)	0.14 (0.14 to 0.15)	0.17 (0.17 to 0.18)
Resistant infections & C. difficile	0.50 (0.49 to 0.51)	0.68 (0.67 to 0.69)	0.63 (0.62 to 0.64)	0.73 (0.72 to 0.74)
Sepsis	0.57 (0.56 to 0.58)	0.65 (0.63 to 0.66)	0.56 (0.55 to 0.57)	0.67 (0.66 to 0.68)
Septic arthritis	0.064 (0.059 to 0.068)	0.064 (0.060 to 0.068)	0.057 (0.053 to 0.061)	0.068 (0.064 to 0.072)

[a]quartiles were estimated from a hierarchical regression model adjusting for age-group, gender, comorbidity, region, deprivation and year

Gulliford MC, *et al. BMJ Open* 2020; 10:e036975. doi: 10.1136/bmjopen-2020-036975

Gulliford et al. Supplementary Data

Supplementary Figure 3: Forest plot showing the adjusted rate ratio for each type of serious bacterial infection for 20% higher total AB prescribing (red) or repeat AB prescribing (grey). Estimates were adjusted for each variable shown and gender, age-group, comorbidity, deprivation fifth, region, year (including quadratic and cubic terms).

■ Acute AB prescribing ● Repeat AB prescribing

	Acute Prescribing				Repeat Prescribing			
**Outcome**	**RR**	**LL**	**UL**	**P value**	**RR**	**LL**	**UL**	**P value**
CNS infection	1.01	0.93	1.09	0.812	0.99	0.96	1.02	0.593
CVS infection	0.94	0.86	1.03	0.186	1.02	0.99	1.06	0.225
Kidney infection	0.98	0.94	1.01	0.197	1.03	1.02	1.05	<0.001
Lung abscess / Empyema	0.92	0.87	0.97	0.003	1.03	1.00	1.05	0.025
Mastoiditis	1.04	0.94	1.15	0.417	1.04	1.00	1.09	0.051
Osteomyelitis	0.96	0.91	1.00	0.055	1.03	1.01	1.05	0.008
Peritonsillar abscess	0.96	0.92	0.99	0.022	1.03	1.02	1.05	<0.001
Resistant infection	1.04	0.98	1.10	0.203	1.00	0.98	1.03	0.752
Sepsis	1.03	0.99	1.07	0.186	1.00	0.98	1.02	0.972
Septic Arthritis	0.94	0.89	0.98	0.007	1.03	1.01	1.05	0.006
**Combined**	**1.02**	**0.99**	**1.05**	**0.227**	**1.01**	**1.00**	**1.02**	**0.054**

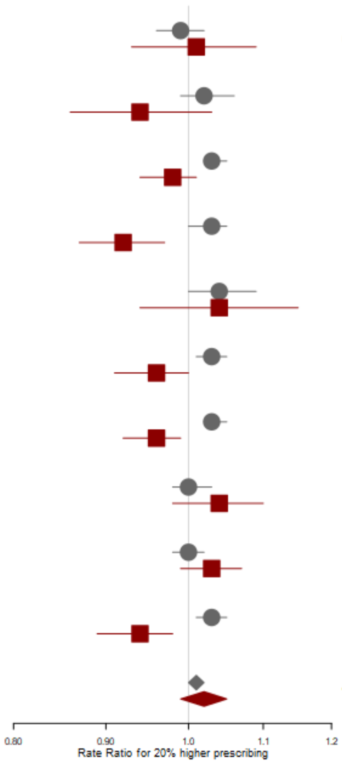

Gulliford MC, *et al. BMJ Open* 2020; 10:e036975. doi: 10.1136/bmjopen-2020-036975
