# Supplementary material for: Serious bacterial infections and antibiotic prescribing in primary care: cohort study using electronic health records in the UK
Source: BMJ Open. 2020 Feb 28;10(2):e036975. doi: 10.1136/bmjopen-2020-036975 (PMC7050314; doi:10.1136/bmjopen-2020-036975)
Supplement: Author's manuscript [file bmjopen-2020-036975.draft_revisions.pdf]

BMJ Open is committed to open peer review. As part of this commitment we make the peer review history of every article we publish publicly available.

When an article is published we post the peer reviewers' comments and the authors' responses online. We also post the versions of the paper that were used during peer review. These are the versions that the peer review comments apply to.

The versions of the paper that follow are the versions that were submitted during the peer review process. They are not the versions of record or the final published versions. They should not be cited or distributed as the published version of this manuscript.

BMJ Open is an open access journal and the full, final, typeset and author-corrected version of record of the manuscript is available on our site with no access controls, subscription charges or pay-per-view fees (http://bmjopen.bmj.com).

If you have any questions on BMJ Open's open peer review process please email info.bmjopen@bmj.com

BMJ Open

SERIOUS BACTERIAL INFECTIONS AND ANTIBIOTIC PRESCRIBING IN PRIMARY CARE. COHORT STUDY USING ELECTRONIC HEALTH RECORDS

Journal:	*BMJ Open*
Manuscript ID	bmjopen-2020-036975
Article Type:	Original research
Date Submitted by the Author:	13-Jan-2020

Complete List of Authors:	Gulliford, Martin; King's College London, UK,<br>Sun, Xiaohui; King's College London, School of Population and Environmental Health Sciences<br>Charlton, Judith; King's College London, UK, Primary Care and Public Health Sciences<br>Winter, Joanne; King's College London, UK<br>Bunce, Catey; kings College london, Primary Care and Public Health Sciences<br>Boiko, Olga; King's College London, UK<br>Fox, Robin; Bicester Health Centre<br>Little, Paul; University of Southampton, Primary Care and Population Science;<br>Moore, Michael; University of Southampton Medical School, Primary Care Medical Group<br>Hay, Alastair; University of Bristol, Centre for Academic Primary Care<br>Ashworth, Mark; King's College London, UK, School of Population Health & Environmental Sciences

Keywords:	PRIMARY CARE, Respiratory infections < THORACIC MEDICINE, Urinary tract infections < UROLOGY, Diagnostic microbiology < INFECTIOUS DISEASES

SCHOLARONE™
Manuscripts

I, the Submitting Author has the right to grant and does grant on behalf of all authors of the Work (as defined in the below author licence), an exclusive licence and/or a non-exclusive licence for contributions from authors who are: i) UK Crown employees; ii) where BMJ has agreed a CC-BY licence shall apply, and/or iii) in accordance with the terms applicable for US Federal Government officers or employees acting as part of their official duties; on a worldwide, perpetual, irrevocable, royalty-free basis to BMJ Publishing Group Ltd ("BMJ") its licensees and where the relevant Journal is co-owned by BMJ to the co-owners of the Journal, to publish the Work in this journal and any other BMJ products and to exploit all rights, as set out in our licence.

The Submitting Author accepts and understands that any supply made under these terms is made by BMJ to the Submitting Author unless you are acting as an employee on behalf of your employer or a postgraduate student of an affiliated institution which is paying any applicable article publishing charge ("APC") for Open Access articles. Where the Submitting Author wishes to make the Work available on an Open Access basis (and intends to pay the relevant APC), the terms of reuse of such Open Access shall be governed by a Creative Commons licence – details of these licences and which Creative Commons licence will apply to this Work are set out in our licence referred to above.

Other than as permitted in any relevant BMJ Author's Self Archiving Policies, I confirm this Work has not been accepted for publication elsewhere, is not being considered for publication elsewhere and does not duplicate material already published. I confirm all authors consent to publication of this Work and authorise the granting of this licence.

For peer review only

SERIOUS BACTERIAL INFECTIONS AND ANTIBIOTIC PRESCRIBING IN PRIMARY CARE. COHORT STUDY USING ELECTRONIC HEALTH RECORDS

Martin C Gulliford MA FRCP,[1,2]	Professor of Public Health
Xiaohui Sun MPH, [1]	PhD student
Judith Charlton MSc,[1]	Research Associate
Joanne R. Winter PhD,[1]	Research Associate
Catey Bunce PhD,[1,2]	Reader in Medical Statistics
Olga Boiko PhD,[1]	Research Associate
Robin Fox MB FRCGP,[3]	General Practitioner
Paul Little MD,[5]	Professor of Primary Care Research
Michael V. Moore BM FRCGP,[5]	Professor of Primary Health Care Research
Alastair D Hay MD,[4]	General Practitioner and Professor of Primary Care
Mark Ashworth DM[1]	Reader in General Practice

And SafeAB Research Group

[1]School of Population Health and Environmental Sciences, King's College London, Guy's Campus, King's College London, London SE1 1UL, UK;
[2]NIHR Biomedical Research Centre at Guy's and St Thomas' Hospitals London, Great Maze Pond, London SE1 9RT, UK;
[3]The Health Centre, Coker Close, Bicester, Oxfordshire, OX26 6AT, UK;
[4]Primary Care Research Group, University of Southampton, Aldermoor Health Centre, Aldermoor Close, Southampton, SO16 5ST, UK;
[5]Centre for Academic Primary Care, Bristol Medical School, Population Health Sciences, University of Bristol, 39 Whatley Rd, Bristol BS8 2PS, UK;

Correspondence: Martin Gulliford
martin.gulliford@kcl.ac.uk
Addison House, Guy's Campus,
King's College London, London SE1 1UL
Tel: 0207 848 6631
Fax: 0207 848 6620

Word count:		
	Text	3,596 words
	Abstract	278 words
	Tables	2
	Figures	3

For peer review only

ABSTRACT

Objective: This study evaluated whether serious bacterial infections are more frequent at family practices with lower antibiotic prescribing rates.
Design: Cohort study.
Setting: 706 UK family practices in the Clinical Practice Research Datalink from 2002 to 2017.
Participants: 10.1 million registered patients with 69.3 million patient-years' follow-up.
Exposures: All antibiotic prescriptions, sub-groups of acute and repeat antibiotic prescriptions, and proportion of antibiotic prescriptions associated with specific-coded indications.
Main outcome measures: First episodes of serious bacterial infections. Poisson models were fitted adjusting for age-group, gender, comorbidity, deprivation, region and calendar year, with random intercepts representing family practice-specific estimates.
Results: The age-standardised antibiotic prescribing rate per 1,000 patient-years increased from 2002 (male 423; female 621) to 2012 (male 530; female 842) before declining to 2017 (male 449; female 753). The median family practice had an antibiotic prescribing rate of 648 per 1,000 patient-years with 95% range for different practices of 430 to 1,038 antibiotic prescriptions per 1,000 patient-years. Specific coded indications were recorded for 58% of antibiotic prescriptions at the median family practice, the 95% range at different family practices was from 10% to 75%. There were 139,759 first episodes of serious bacterial infection. After adjusting for covariates and the proportion of coded consultations, there was no evidence that serious bacterial infections were lower at family practices with higher total antibiotic prescribing. The adjusted rate ratio (RR) for 20% higher total antibiotic prescribing was 1.03, (95% confidence interval 1.00 to 1.06, P=0.074).
Conclusions: We did not find population-level evidence that family practices with lower total antibiotic prescribing might have more frequent occurrence of serious bacterial infections overall. Improving the recording of infection episodes has potential to inform better antimicrobial stewardship in primary care.

Key words: antibiotics; primary care; respiratory tract infections; peritonsillar abscess; mastoiditis.

[278 words]

For peer review only

Strengths and limitations of this study

- This cohort study included 10.1 million patients with 69.3 million patient-years of follow-up at 706 UK family practices from 2002 to 2017.
- The study included all antibiotic prescriptions and classified them according to the medical conditions recorded on the same date
- The study relied on medical conditions recorded by health care professionals in primary care
- Missing and misclassified information might result in bias, which might generally be towards a null finding
- The study aimed to evaluate associations at the general practice-level and the results do not exclude the possibility of association at the individual patient-level

For peer review only

INTRODUCTION

Antimicrobial resistance is a growing concern for health systems. The G20 health ministers noted that ‘drug-resistant [organisms] are to blame for 700,000 deaths worldwide each year, and this figure is predicted to rise to 10 million by 2050 if urgent action is not taken.’ (1) There are now intense efforts to reduce unnecessary use of antibiotics, especially in primary care where 80% of antibiotics are prescribed. These antimicrobial stewardship programmes have met with some success. In England, the total quantity of antibiotics prescribed in primary care declined by 13.2% in the 5 years between 2013 and 2017.(2, 3) Bacterial infections are still of public health importance with 1.7 million cases of sepsis and 270,000 deaths per year in the U.S.(4) Strategies to reduce inappropriate use of antibiotics must ensure that antibiotics can be used when they are needed.(5, 6)

It is possible that reducing antibiotic prescribing might be associated with greater risk of serious bacterial infections. Previous research investigated infection risk and antibiotic prescribing for respiratory illnesses.(3, 7) In a cohort study, Petersen et al.(8) found that antibiotic treatment reduced risks of mastoiditis after otitis media, peritonsillar abscess after sore throat, and pneumonia after respiratory infection. An analysis of electronic health records,(9) found that family practices that prescribed antibiotic more frequently to patients with self-limiting respiratory illnesses might have lower risk of pneumonia and peritonsillar abscess but there were no associations with risk of mastoiditis, empyema, meningitis, intracranial abscess or Lemierre’s syndrome. A cluster- randomised trial of an antimicrobial stewardship intervention for respiratory prescribing,(10) as well as an interrupted time series analysis found no clear evidence that antimicrobial stewardship policies might be associated with increased bacterial infections overall.(11) However, Gharbi et al.(12) found that apparent non-use of antibiotics for urinary infections might be associated with higher risk of sepsis.

For peer review only

It is important to extend these investigations to include antibiotic prescribing for all indications because the reasons for antibiotic prescribing may not always be well-documented, with up to half of antibiotic prescriptions in UK primary care not associated with any record of specific diagnostic medical codes.(3, 7) When analyses are restricted to antibiotic prescriptions for clearly recorded indications, the true extent of antibiotic prescribing may be under-estimated. It is also important to assess repeat antibiotic prescriptions which may be given for prevention of recurrent infections or treatment of serious or chronic infections.(3) The present study aimed to test the hypothesis that greater use of antibiotics for all indications might be associated with lower risk of serious bacterial infection. We also investigated whether patterns of medical coding were associated with the apparent occurrence of serious bacterial infection.

For peer review only

For peer review only - http://bmjopen.bmj.com/site/about/guidelines.xhtml

METHODS

Data source

We carried out a population-based cohort study in the UK Clinical Practice Research Datalink (CPRD) employing data for 2002 to 2017. The CPRD is one of the world's largest databases of primary care electronic health records, with participation of about 7% of UK family practices and with ongoing collection of anonymised data from 1990.(13) The high quality of CPRD data has been confirmed in many studies.(14) We ascertained serious bacterial infection events from the entire population of CPRD because these are generally rare events. In order to estimate family practice-level prescribing metrics, we analysed a sample of CPRD data because it was not feasible to analyse all antibiotic prescribing in the whole of CPRD. The protocol for the study has been published. The protocol was approved by the CPRD Independent Scientific Advisory Committee (ISAC protocol 18-041R).

Selection of sample for antibiotic prescribing analysis

In order to analyse antibiotic prescribing, a sample was drawn from the CPRD denominator file for the October 2018 release of CPRD. A random sample of registered patients was drawn, stratifying by year between 2002 and 2017 and by family practice. In each year of study, a sample of 10 participants was taken for each gender and age group using five-year age groups up to a maximum of 104 years. Each sampled participant contributed data in multiple years of follow-up. There was a total sample of 671,830 individual participants, registered at a total of 706 family practices, who contributed person time between 2002 and 2017. The sampling design enabled estimation of all age-specific rates with similar precision, while age-standardisation provided weightings across age groups.

For peer review only - http://bmjopen.bmj.com/site/about/guidelines.xhtml

Main measures for antibiotic prescribing

For each participant in the antibiotic prescribing sample, we calculated the person-time at risk between the start and end of the patient's record. Person time was grouped by gender, age-group and comorbidity. Age groups were from 0 to 4, 5 to 9 and 10 to 14 and then 10-year age groups up to 85 years and over. Comorbidity was evaluated as either present or absent in each person-year using the 'seasonal flu at risk codes' which are used to identify individuals at higher risk of infection who may benefit from influenza vaccination.(15) Seasonal flu at risk Read codes include medical diagnostic codes for overweight and obesity, coronary heart disease, chronic kidney disease, chronic liver disease, chronic neurological disease, chronic respiratory disease, diabetes mellitus and disorders of the immune system and drug product codes for asthma therapy, corticosteroid drugs and immunosuppressive drugs.

Antibiotic prescriptions were evaluated using product codes for antibiotics listed in section 5.1 of the British National Formulary, excluding methenamine and drugs for tuberculosis, and leprosy. Different antibiotic classes and antibiotic doses were not considered further in this analysis. Multiple antibiotic prescription records on the same day were considered as a single antibiotic prescription. Medical codes recorded on the same date as the antibiotic prescription were used to classify the indication for prescription using categories of 'respiratory', 'genito-urinary', 'skin', and 'other specific' indications. All other codes were classified as 'non-specific' codes. (3) A prescriptions was classified as 'acute' if it was the first prescription in a sequence or 'repeat' prescription otherwise, as reported previously.(3) Antibiotic prescriptions that were not associated with medical codes and were not repeat prescriptions were classified as 'no codes recorded'.

For peer review only - http://bmjopen.bmj.com/site/about/guidelines.xhtml

Serious bacterial infections

Incident cases of serious bacterial infection were evaluated in the January 2019 release of CPRD for the years 2002 to 2017 with the CPRD denominator providing the person time at risk. The mean duration of follow-up was 6.9 years. Serious bacterial infections were selected for study from review of the International Classification of Diseases 10th revision,(16) the Read code classification(17) and through discussion with the research team. The final list of conditions is summarised in Table 1 and included: bacterial infections of the central nervous system (CNS); bacterial infections of the cardiovascular system (CVS); kidney infections; lung abscess and empyema; mastoiditis; osteomyelitis; peritonsillar abscess; resistant infections and C. difficile; sepsis and septic arthritis. Incident events were first records for each type of serious bacterial infection in a patient more than 12 months after the start of the patient record. Possible recurrent events in the same patient were not evaluated further because, in electronic health records, it may not be possible to distinguish new occurrences from reference to ongoing or previous problems.

Statistical analysis

The analysis was in two stages. First, we estimated family practice-specific estimates for antibiotic prescribing; secondly, we evaluated whether these estimates were associated with the risk of serious bacterial infection. In the first stage of the analysis, we analysed antibiotic prescribing in primary care between 2002 and 2017 (Supplementary Table 1: Model 1). A hierarchical Poisson model was fitted using the 'hglm' package in the R program,(18) with counts of antibiotic prescriptions as the outcome and the log of person time as the offset. Estimates were adjusted for the fixed effects of gender, age-group, fifth of deprivation at family practice-level, comorbidity, and region in the UK. Calendar year was included as a continuous predictor together with quadratic and cubic terms to allow for non-linear trends. Random intercepts were estimated for each family practice and each estimate represented the adjusted log relative rate for antibiotic prescribing at that practice compared with the

For peer review only - http://bmjopen.bmj.com/site/about/guidelines.xhtml

overall mean. The proportion of antibiotic prescriptions that were associated with specific medical codes was analysed in a similar framework with coded prescriptions as the outcome and the log of antibiotic prescriptions as the offset.

In the second stage of analysis, serious bacterial infections were analysed as the outcome (Supplementary Table 1: Model 2). The antibiotic prescribing level for each family practice was included as a predictor using the family practice-specific estimates from Model 1. These estimates initially had a mean of zero and standard deviation of 0.19, consistent with an adjusted relative rate of antibiotic prescribing of 1.21 for a family practice with prescribing one standard deviation above the mean. Estimates were standardised to give the change in serious bacterial infection for a 20% relative increase in antibiotic prescribing rate at a practice. A 20% change represents a clinically important change in antibiotic prescribing. We also estimated the change in serious bacterial infection for a 20% relative increase in proportion of antibiotic prescriptions with specific medical codes recorded at a family practice. Models were adjusted for age-group, gender, region, deprivation fifth, calendar year (including quadratic and cubic terms for the latter), with log of person-time as offset. The results were visualised using forest plots.(19)

For peer review only - http://bmjopen.bmj.com/site/about/guidelines.xhtml
For peer review only

RESULTS

There were 706 family practices included in the analysis, with 10.1 million registered patients and 69.3 million patient years of follow-up. In the sub-sample analysed for antibiotic prescribing, there were 706 family practices with 6,541,195 person-years of follow-up (Supplementary Figure 1 and Supplementary Table 2). There was a total of 4,371,715 antibiotic prescriptions between 2002 and 2017. This included 2,368,551 (54%) with coded indications including 1,531,645 (35%) associated with respiratory infections, 369,389 (8%) with genitourinary infections, 414,680 (10%) with skin infections and 52,837 (1%) with other specific indications. There were 2,003,164 (46%) of antibiotic prescriptions without specific coded indications consisting of 479,421 (11%) repeat prescriptions, 1,154,789 (26%) with non-specific medical codes recorded and 368,954 (8%) with no medical codes recorded.

Supplementary Figure 2 shows changes over time in age-standardised antibiotic prescribing rates per 1,000 patient years for coded and not coded indications. During the initial period of the study from 2002 to 2012, the age-standardised total antibiotic prescribing rate per 1,000 patient years increased from 2002 (male 423; female 621) to 2012 (male 530; female 842) before declining to 2017 (male 449; female 753). The recent decrease in total antibiotic prescribing was accompanied by a decline in antibiotic prescribing for coded indications, but antibiotic prescriptions that were not associated with specific coded indications continued to increase. There was evidence of a decline in antibiotic prescribing for respiratory illness from 2008 onwards (Figure 1) and after 2012 there was evidence of decreasing prescribing for genito-urinary and skin infections, as well as other specific indications. Throughout the period from 2002 to 2017, antibiotic prescriptions associated with non-specific codes increased as did repeat prescriptions. Antibiotic prescriptions that were not associated with medical codes declined initially but then remained constant (Figure 1).

For peer review only - http://bmjopen.bmj.com/site/about/guidelines.xhtml

Table 2 summarises variation in antibiotic prescribing metrics between family practices in the sample. The 95% range for family practice-specific antibiotic prescribing rates was from 430 to 1,038 antibiotic prescriptions per 1,000 person-years, with a median of 648 antibiotic prescriptions per 1,000 patient years. The 95% range for the proportion of repeat prescriptions was from 3% to 24%. The 95% range for the proportion of antibiotic prescriptions with specific coded indications recorded ranged from 10% to 75%.

There were 139,759 first episodes of serious bacterial infections (Supplementary Table 3). Figure 2 shows trends in the age-standardised incidence of serious bacterial infections from 2002 to 2017. The total incidence of serious bacterial infections increased during the period. This increase was largely accounted for by increases in sepsis, antibiotic resistant and C. difficile infections, kidney infections and osteomyelitis. The remaining conditions showed either stable incidence or slight declines. Supplementary Table 4 presents age- and sex-standardised incidence rates per 1,000 patient-years for serious bacterial infections for the highest and lowest fourths of antibiotic prescribing. There was no evidence that serious bacterial infections might be more frequent at family practices in the lowest fourth of antibiotic prescribing. In general, age- and sex-standardised incidence rates tended to be highest at family practices that were higher prescribers of antibiotics. Supplementary Table 4 also compares the incidence of serious bacterial infection for the lowest and highest fourths of medical coding. Family practices in the highest fourth of medical coding had an incidence of serious bacterial infection of 2.39 per 1,000 patient years (95% confidence interval 2.37 to 2.42) compared with 1.94 (1.91 to 1.96) in the lowest fourth of medical coding.

Figure 3 presents a forest plot for the association of each serious bacterial infection with 20% higher total antibiotic prescribing at a family practice. The combined estimate revealed that there was no evidence that higher total antibiotic prescribing was associated with lower

For peer review only - http://bmjopen.bmj.com/site/about/guidelines.xhtml
For peer review only

incidence of serious bacterial infections (adjusted rate ratio 1.03, 95% confidence interval 1.00 to 1.06, P=0.074). When the 10 classes of serious bacterial infection were considered individually, there was no evidence that higher antibiotic prescribing might be associated with a lower incidence of infections. However, there was weak evidence of that lung abscess and empyema (RR 0.94, 0.88 to 1.00, P=0.038) might be lower at higher prescribing family practices. There was strong evidence that the recorded incidence of serious bacterial infections was associated with the coding of specific indications for a antibiotic prescriptions (adjusted rate ratio for a 20% increase in coding proportion 1.24, 1.18 to 1.29, P<0.001). This association held for each of the 10 classes of serious bacterial infections considered individually.

We conducted a sensitivity analysis by excluding repeat prescriptions that might not have been for acute infection episodes. There was no evidence that higher acute (non-repeat) antibiotic prescribing was associated with serious bacterial infections overall (RR 1.02, 0.99 to 1.05, P=0.227). (Supplementary Figure 3) There was evidence that higher acute antibiotic prescribing might be associated with lower incidence of lung abscess and empyema and septic arthritis. Osteomyelitis and peritonsillar abscess were not judged to be associated with acute antibiotic prescribing after controlling the false discovery rate. There was weak evidence that higher repeat antibiotic prescribing might be associated with higher incidence of serious bacterial infections overall (RR 1.01, 1.00 to 1.02, P=0.054) with evidence of this association for kidney infections, osteomyelitis, peritonsillar abscess and septic arthritis considered separately.

For peer review only - http://bmjopen.bmj.com/site/about/guidelines.xhtml

DISCUSSION

Principal findings

This study found that antibiotic prescribing increased from 2002 to 2012 but declined subsequently with changes over time being of larger magnitude for women than men. The incidence of serious bacterial infections in men and women rose steadily between 2002 and 2017, particularly for sepsis (men and women), osteomyelitis (mainly in men), and kidney infections (mainly in women). The research aimed to test the hypothesis that family practices with lower utilisation of antibiotics might have greater risk of serious bacterial infections. We evaluated the incidence of serious bacterial infections including 10 groups of infections that affect different systems of the body as well as sepsis (including septicaemia). We did not find evidence that family practices that prescribe antibiotics less frequently might have a higher incidence of serious bacterial infections. We found evidence that each type of serious bacterial infection was recorded more frequently at family practices that record diagnostic codes for a high proportion of antibiotic prescriptions suggesting that variation in the incidence of serious bacterial infection among family practices may be partly an artefact of data-recording. Measures are needed to improve the recording of infection episodes in primary care both when antibiotics are prescribed and when they are not. Repeat prescriptions account for a significant proportion of uncoded prescriptions (3) and repeat prescriptions might be indicated for prolonged or serious infections. We conducted analyses after excluding repeat prescriptions and these analyses raised the possibility that family practices with lower acute (non-repeat) antibiotic prescribing might have higher incidence of lung abscess and empyema and septic arthritis. However, these analyses were not pre-planned, should be considered as hypothesis-generating and requiring confirmation in future studies. The incidence of these two conditions is less than one per 10,000 patients per year, and a relative rate of 0.9 for a 20% increase in prescribing implies that at most one additional case might arise every 10 years from a 20% reduction in prescribing at a family practice with 10,000 registered patients.

For peer review only - http://bmjopen.bmj.com/site/about/guidelines.xhtml

Strengths and weaknesses of the study

The study drew on data for a large population comprising data for about 7% of the UK general population. In view of sample size constraints, antibiotic utilisation was estimated through analysis of data for a sample of patients, using hierarchical (multilevel) regression models to obtain family practice-specific antibiotic prescribing estimates. This contrasts with our previous study in which age- and sex-standardised rates were calculated from the data for each practice.(9) Use of a regression modelling approach enabled us to make optimal use of the data, as well as adjusting for covariates that are associated with variations in antibiotic prescribing (20) including comorbidity, deprivation, region and calendar year, in addition to age and sex.(21) Consistent with previous studies,(3, 7) we observed that nearly half of antibiotic prescriptions were not associated with specific coded indications. This suggests that total antibiotic prescribing is the most appropriate exposure measure for consideration, because indication-specific antibiotic prescribing may be associated with considerable misclassification. Serious bacterial infections were identified from medical diagnostic codes recorded into primary care electronic health records. Many studies have shown that these records have a high predictive value for a range of diagnoses, (14) but relying on a single data source can lead to under-estimation of the total number of events.(22) There may be changes over time in the use of diagnostic categories, which might in part account for increasing diagnoses of 'sepsis'. A study of U.S. hospitals' data found that there was a 706% increase in sepsis between 2003 and 2012, without any corresponding increase in positive blood cultures.(23) An interrupted time series analysis,(11) offers an alternative approach to analysis but this might be susceptible to changes over time in unmeasured confounders such as code selection. The results of our study draw attention to the problem of poor coding quality in the context of infection management in primary care. Evidence from other studies suggests that missing values are typically missing not at random and the act of data recording may introduce a form of confounding by indication that may bias results.(24) In order to allow for this, we explicitly evaluated the extent to which differences in data recording between practices might account

For peer review only - http://bmjopen.bmj.com/site/about/guidelines.xhtml

for variations in the incidence of serious bacterial infections. It is likely that misclassification of exposure and outcome variables, from incomplete data recording, might lead to under-estimation of associations, though the direction of bias cannot always be anticipated.(25) We adjusted for a summary measure of comorbidity. Our analyses do not exclude the possibility that there may be vulnerable sub-groups of patients, such as those with immunosuppression, who may be at increased risk if antibiotics are withheld.

Comparison with other studies

The trends in total antibiotic utilisation reported here are consistent with national trends based on aggregate data.(2) Neilly et al.(26) found that increasing prescription volumes in the period up to 2013 could be accounted for by increasing dose and duration of prescriptions but we found evidence of increased antibiotic prescribing based on numbers of prescriptions alone. Consistent with our findings, Balinskaite et al.(11) reported increasing rates of infection in English primary care and hospital admissions data from 2010 to 2017. Their time series analysis suggested that antimicrobial stewardship intervention in 2015 had no impact on bacterial infections overall but there was some evidence for increasing hospital admissions for quinsy, decreasing hospital admissions for pyelonephritis and decreasing GP consultation rates for empyema. In a previous study, we found that peritonsillar abscess and pneumonia might be more frequent when family practices prescribe antibiotics less frequently for respiratory tract infections.(9) We did not include pneumonia in this study because we found that syndromes of ‘chest infection’ and ‘pneumonia‘ may be difficult to distinguish in primary care records with evidence of code shifting between the two categories.(27) In the present study, the incidence of peritonsillar abscess was not associated with total antibiotic prescribing. Randomised trials suggest that antibiotics protect against peritonsillar abscess (28) so it is plausible that this condition might be associated with respiratory antibiotic prescribing but not total antibiotic prescribing.

For peer review only - http://bmjopen.bmj.com/site/about/guidelines.xhtml

Patient and public involvement

The protocol and results of the study were discussed at meetings with patients. Patients commented on the recent declining trend in antibiotic prescribing. They noted that avoiding antibiotics requires trade-offs between the limited benefits from antibiotic treatment, the side effects of antibiotic use, and the potential from longer-term problems from the increase in antimicrobial resistance. Patients considered that risks of serious bacterial infections were generally low at the present time. There is a need to communicate these results to patients and prescribers so that both groups can be aware of the wider contextual issue of antimicrobial resistance to inform antibiotic prescribing decisions.

Main conclusions

Family practices that reduce the amount of antibiotics prescribed do not risk any increase in serious bacterial infections overall. This finding does not exclude the possibility that serious bacterial infection may be associated with antibiotic prescribing patterns at individual patient-level. Consequently, reducing antibiotic utilisation in primary care will require a detailed understanding of when antibiotics prescriptions are required and when they are not and increasing the quality of data recording with respect to antibiotic use should be a high priority. This study focused on population-level associations at the level of family practice. Future research should evaluate the associations at the level of the individual patient and the individual family practice consultation.

For peer review only - http://bmjopen.bmj.com/site/about/guidelines.xhtml

Acknowledgement

The SafeABStudy Group also includes Dr Caroline Burgess, Dr Vasa Curcin and Dr James Shearer.

Data sources

The study is based in part on data from the Clinical Practice Research Datalink obtained under license from the UK Medicines and Healthcare products Regulatory Agency. However, the interpretation and conclusions contained in this report are those of the authors alone.

Data sharing

Requests for access to data from the study should be addressed to martin.gulliford@kcl.ac.uk. The study protocol has been published. All proposals requesting data access will need to specify planned uses with approval of the study team and CPRD before data release.

Funding

The study is funded by the National Institute for Health Research (NIHR) Health Services and Delivery Programme (16/116/46). MG was supported by the NIHR Biomedical Research Centre at Guy's and St Thomas' Hospitals. The views expressed are those of the authors and not necessarily those of the NHS, the NIHR, or the Department of Health. The funder of the study had no role in study design, data collection, data analysis, data interpretation, or writing of the report. The authors had full access to all the data in the study and all authors shared final responsibility for the decision to submit for publication.

Conflict of Interest

The authors have no conflicts of interest.

Author Contributions

MG wrote the study protocol with advice from CB, RF, MA, PL, MM and AH; XS developed and piloted code sets and analyses for antibiotic prescribing; RF, PL, MM, AH and MA reviewed case definitions; JC programmed analyses and JW advised; MG completed data analyses and drafted the paper with advice from CB, RF, PL, MM, AH and MA; OB coordinated PPI input. All authors reviewed and contributed to the final draft. MG is guarantor.

For peer review only

For peer review only - http://bmjopen.bmj.com/site/about/guidelines.xhtml

REFERENCES

1. G20 Information Centre. *Declaration: G20 Meeting of Health Ministers 2018* [Available from: http://www.g20.utoronto.ca/2018/2018-10-04-health.html accessed 11th October 2019]

2. Public Health England. *English Surveillance Programme for Antimicrobial Utilisation and Resistance (ESPAUR) Report 2017*. London: Public Health England, 2017. [Available from https://assets.publishing.service.gov.uk/government/uploads/system/uploads/attachment_data/file/759975/ESPAUR_2018_report.pdf accessed 11th October 2019]

3. Sun X, Gulliford MC. Reducing antibiotic prescribing in primary care in England from 2014 to 2017: population-based cohort study. *BMJ open*. 2019;**9** (7):e023989.

4. Centers for Disease Control and Prevention. *Sepsis: Data and Reports.* Atlanta, GA: Centers for Disease Control and Prevention, 2019: Source: https://www.cdc.gov/sepsis/datareports/index.html accessed 28th October 2019.

5. Laxminarayan R, Matsoso P, Pant S, Brower C, Røttingen J-A, Klugman K, et al. Access to effective antimicrobials: a worldwide challenge. *The Lancet.* 2016; **387**(10014):168-75.

6. NHS England. *Quality Premium: 2016/17 Guidance for CCGs.* Leeds: NHS England, 2016.

7. Dolk FCK, Pouwels KB, Smith DRM, Robotham JV, Smieszek T. Antibiotics in primary care in England: which antibiotics are prescribed and for which conditions? *J Antimicrobial Chemother*. 2018;**73** (suppl_2):ii2-ii10.

8. Petersen I, Johnson AM, Islam A, Duckworth G, Livermore DM, Hayward AC. Protective effect of antibiotics against serious complications of common respiratory tract

For peer review only - http://bmjopen.bmj.com/site/about/guidelines.xhtml

infections: retrospective cohort study with the UK General Practice Research Database. *BMJ*. 2007; **335**: 982.

9. Gulliford MC, Moore MV, Little P, Hay AD, Fox R, Prevost AT, et al. Safety of reduced antibiotic prescribing for self limiting respiratory tract infections in primary care: cohort study using electronic health records. *BMJ* 2016;**354**:i3410.

10. Gulliford MC, Prevost AT, Charlton J, Juszczyk D, Soames J, McDermott L, et al. Effectiveness and safety of electronically delivered prescribing feedback and decision support on antibiotic use for respiratory illness in primary care: REDUCE cluster randomised trial. *BMJ*. 2019;**364**:l236.

11. Balinskaite V, Aylin P, Johnson AP, Holmes A. The Impact of a National Antimicrobial Stewardship Program on Antibiotic Prescribing in Primary Care: An Interrupted Time Series Analysis. *Clin Infect Dis.* 2019; **69** (2):233-242. doi: 10.1093/cid/ciy904.

12. Gharbi M, Drysdale JH, Lishman H, Goudie R, Molokhia M, Johnson AP, et al. Antibiotic management of urinary tract infection in elderly patients in primary care and its association with bloodstream infections and all cause mortality: population based cohort study. *BMJ.* 2019;**364**:l525.

13. Herrett E, Gallagher AM, Bhaskaran K, Forbes H, Mathur R, van Staa T, et al. Data Resource Profile: Clinical Practice Research Datalink (CPRD). *Int J Epidemiol*. 2015;**44** (3)**:** 827-36.

14. Herrett E, Thomas SL, Schoonen WM, Smeeth L, Hall AJ. Validation and validity of diagnoses in the General Practice Research Database: a systematic review. *Br J Clin Pharmacol.* 2010;**69** (1):4-14.

15. NHS Employers. *Seasonal flu at risk Read Codes 2015-2016*. Leeds: NHS Employers, 2016.

For peer review only - http://bmjopen.bmj.com/site/about/guidelines.xhtml

16. World Health Organization. *International Statistical Classification of Diseases and Related Health Problems 10th Revision 2010* [Available from: http://apps.who.int/classifications/icd10/browse/2010/en accessed 11th October 2019.

17. NHS Digital. *Clinical terms (Read codes). Summarised product description*. Leeds: NHS Digital, 2018. Available at https://isd.digital.nhs.uk/trud3/user/guest/group/0/pack/9/subpack/19/releases accessed 11th October 2019.

18. Lee Y, Ronnegard L, Noh M. *Data analysis using hierarchical generalized linear models with R.* Boca Raton, FL: CRC Press; 2017.

19. Gordon M, Lumley T. *Advanced Forest Plot Using 'grid' Graphics.* Vienna: The Comprehensive R Archive Network, 2016. Source: https://cran.r-project.org/web/packages/forestplot/forestplot.pdf. accessed 11th October 2019.

20. Pouwels KB, Dolk FCK, Smith DRM, Smieszek T, Robotham JV. Explaining variation in antibiotic prescribing between general practices in the UK. *J Antimicrobial Chemother* 2018;**73** (suppl_2):ii27-ii35.

21. Goldstein H, Spiegelhalter DJ. League Tables and Their Limitations: Statistical Issues in Comparisons of Institutional Performance. *J Royal Statistical Society, A*. 1996;**159** (3) :385-443.

22. Herrett E, Shah AD, Boggon R, Denaxas S, Smeeth L, van Staa T, et al. Completeness and diagnostic validity of recording acute myocardial infarction events in primary care, hospital care, disease registry, and national mortality records: cohort study. *BMJ.* 2013;**346**:f2350.

For peer review only - http://bmjopen.bmj.com/site/about/guidelines.xhtml

23. Rhee C, Murphy MV, Li L, Platt R, Klompas M. Comparison of trends in sepsis incidence and coding using administrative claims versus objective clinical data. *Clin infectious dis* 2015;**60**(1)**:**88-95.

24. Agniel D, Kohane IS, Weber GM. Biases in electronic health record data due to processes within the healthcare system: retrospective observational study. *BMJ*. 2018;**361:** k1479.

25. Greenland S, Robins JM. Confounding and misclassification. *Am J Epidemiol.* 1985;**122** (3):495-506.

26. Neilly MDJ, Guthrie B, Hernandez Santiago V, Vadiveloo T, Donnan PT, Marwick CA. Has primary care antimicrobial use really been increasing? Comparison of changes in different prescribing measures for a complete geographic population 1995–2014. *J Antimicrobial Chemother*. 2017;**72** (10)**:**2921-30.

27. Sun X, Douiri A, Gulliford M. Pneumonia incidence trends in UK primary care from 2002 to 2017: population-based cohort study. *Epidemiol Infect.* 2019;**147**:e263. doi: 10.1017/S0950268819001559.

28. Spinks A, Glasziou PP, Del Mar CB. Antibiotics for sore throat. *Cochrane database syst rev*. 2013;**11**:CD000023.

For peer review only

For peer review only - http://bmjopen.bmj.com/site/about/guidelines.xhtml

Table 1: Groups of serious bacterial infections including numbers of medical codes and five most frequently recorded conditions. Figures are frequencies.

Group	Number of codes	Number of first events	Five most frequent conditions (number of first events 2002 to 2017)
CNS Infection	30	576	Epidural abscess (117), cerebral abscess (112), brain abscess (79), intraspinal abscess (49), drainage of abscess of subdural space (44)
CVS infection	24	1,697	Acute and subacute endocarditis (594), bacterial endocarditis (276), Subacute bacterial endocarditis (270), acute endocarditis NOS (166), acute bacterial endocarditis (114)
Kidney Infection	22	30,827	Acute pyelonephritis (19.284), pyelonephritis unspecified (7,115), infections of kidney (1,670), acute pyelitis (1,008), pyelitis unspecified (745)
Lung abscess / empyema	24	2,932	Empyema (2,314), abscess of lung (149), abscess of lung and mediastinum (139), thorax abscess NOS (68), pleural empyema (56)
Mastoiditis	10	1,970	Mastoiditis and related conditions (1,293), mastoiditis NOS (487), acute mastoiditis (146), acute mastoiditis NOS (31), abscess of mastoid (27)
Osteomyelitis	65	4,921	Acute osteomyelitis (3,297), unspecified osteomyelitis (678), unspecified osteomyelitis of unspecified site (284), osteomyelitis jaw (78), unspecified osteomyelitis NOS (75)
Peritonsillar abscess	6	11,338	Quinsy (8,611), peritonsillar abscess – quinsy (1,748), O/E quinsy present (654), drainage of peritonsillar abscess (232), drainage of quinsy (226),
Resistant infections & C. difficile	31	42,185	Clostridium difficile toxin detection (20,175), methicillin resistant staphylococcus aureus positive (9,914), Clostridium difficile infection (6,397), methicillin resistant staphylococcus aureus (4,303), methicillin resistant staphylococcus aureus carrier (1,017)
Sepsis	100	39,059	Sepsis (23,149), septicaemia (6,204), urosepsis (4,646), biliary sepsis (1,233), Clostridium infection (576)
Septic arthritis	41	4,254	Septic arthritis (3,649), Pyogenic arthritis (184), Arthropathy associated with infections (172), Knee pyogenic arthritis (52), Staphylococcal arthritis and polyarthritis (39)

For peer review only - http://bmjopen.bmj.com/site/about/guidelines.xhtml

Table 2: Variation in antibiotic prescribing between family practices. Figures represent the centiles of the distribution of family practice-specific values.

	Centiles of family practices				
**Measure**	**2.5th**	**25th**	**Median**	**75th**	**97.5th**
AB prescribing rate per 1,000 patient-years	430	563	648	748	1,038
Acute prescriptions (% of all antibiotic prescriptions)	76	86	90	93	97
Repeat prescriptions (% of all antibiotic prescriptions)	3	7	10	14	24
Coded indication (% of all antibiotic prescriptions)	10	48	58	65	75
Respiratory (% of all antibiotic prescriptions)	6	31	36	42	52
Genito-urinary (% of all antibiotic prescriptions)	1	7	8	11	16
Skin (% of all antibiotic prescriptions)	2	8	10	12	16
Other specific (% of all antibiotic prescriptions)	0	1	1	2	3
Non-coded indications (% of all antibiotic prescriptions)	24	35	42	51	90
No codes recorded (% of all antibiotic prescriptions)	1	3	6	11	28
Non-specific codes recorded (% of all antibiotic prescriptions)	12	19	24	29	59

Column percents are not expected to sum to 100 as different family practices may be represented for the same centile in different rows

For peer review only - http://bmjopen.bmj.com/site/about/guidelines.xhtml

Figure 1: Age- and sex-standardised antibiotic prescribing rates per 1,000 patient years for coded and not coded indications from 2002 to 2017.

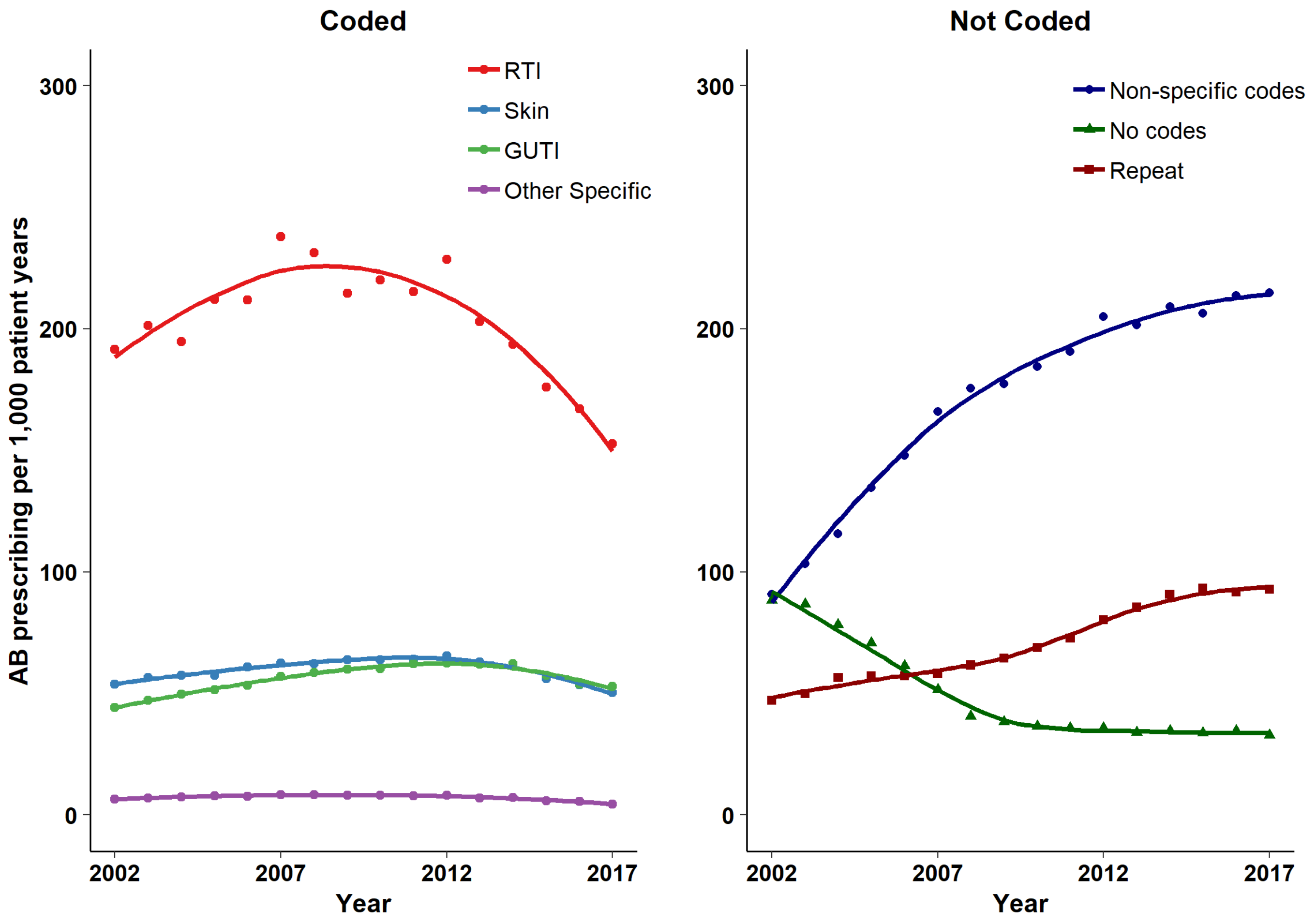

Figure 2: Age-standardised rates of serious bacterial infections per 1,000 patient years from 2002 to 2017. Red lines, female; blue lines, male; shaded areas, 95% confidence intervals.

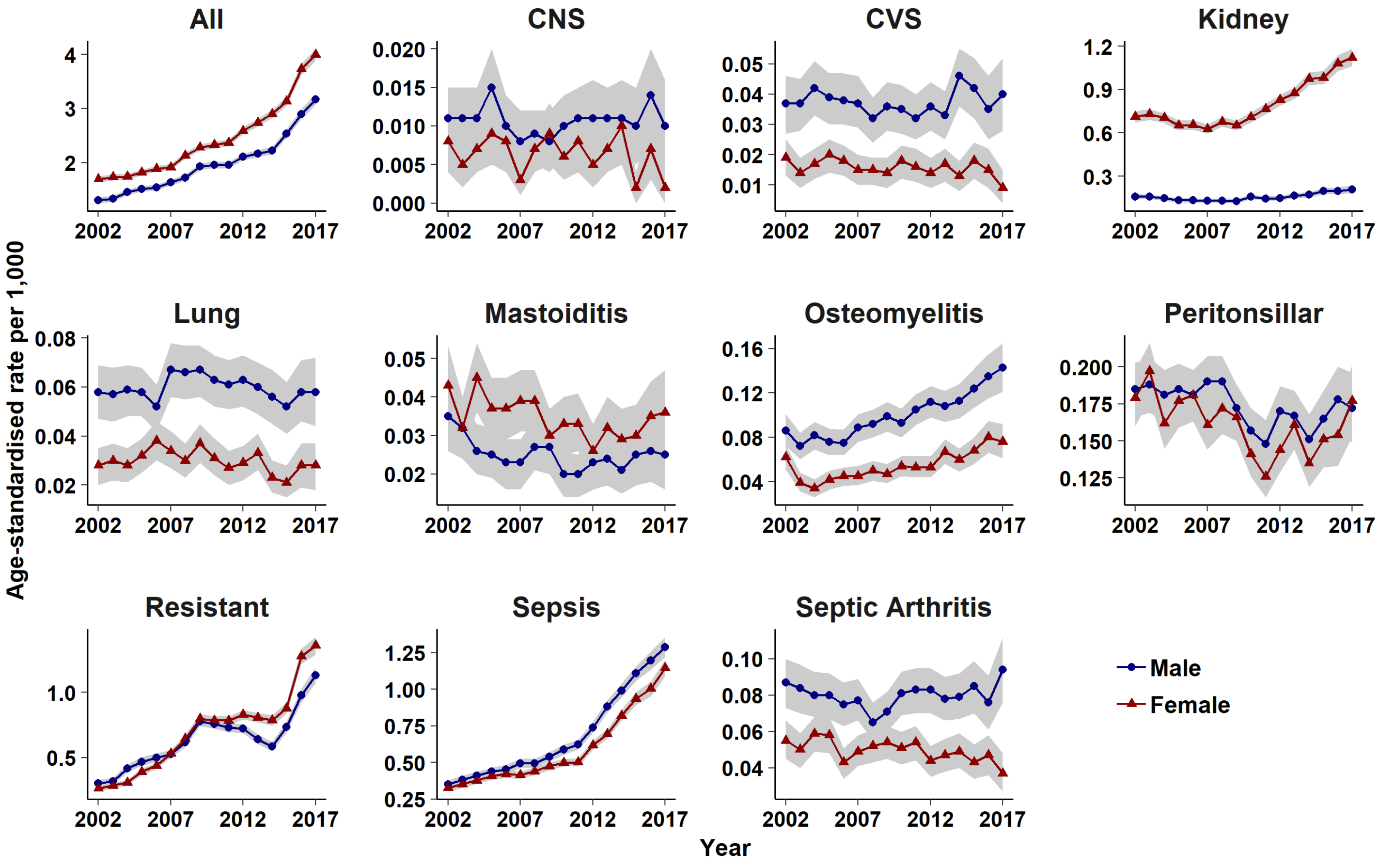

Figure 3: Forest plot showing the adjusted rate ratio for each type of serious bacterial infection for 20% higher total antibiotic prescribing (red) or 20% higher proportion of antibiotic prescriptions with specific coded indications recorded (grey). Estimates were adjusted for each variable shown and gender, age-group, comorbidity, deprivation fifth, region and year (including quadratic and cubic terms).

	All AB Prescribing				Medical Coding			
**Outcome**	**RR**	**LL**	**UL**	**P value**	**RR**	**LL**	**UL**	**P value**
CNS infection	1.01	0.93	1.10	0.853	1.14	1.01	1.30	0.039
CVS infection	0.96	0.87	1.06	0.402	1.22	1.06	1.41	0.005
Kidney infection	1.00	0.96	1.04	0.921	1.28	1.20	1.35	0.000
Lung abscess / Empyema	0.94	0.88	1.00	0.038	1.15	1.05	1.25	0.002
Mastoiditis	1.08	0.97	1.20	0.179	1.52	1.29	1.79	0.000
Osteomyelitis	0.97	0.93	1.02	0.287	1.14	1.06	1.23	0.001
Peritonsillar abscess	0.98	0.94	1.02	0.334	1.21	1.14	1.28	0.000
Resistant infection	1.04	0.98	1.11	0.161	1.38	1.26	1.51	0.000
Sepsis	1.03	0.99	1.08	0.185	1.23	1.15	1.31	0.000
Septic Arthritis	0.96	0.91	1.01	0.123	1.23	1.14	1.33	0.000
**Combined**	**1.03**	**1.00**	**1.06**	**0.074**	**1.24**	**1.18**	**1.29**	**0.000**

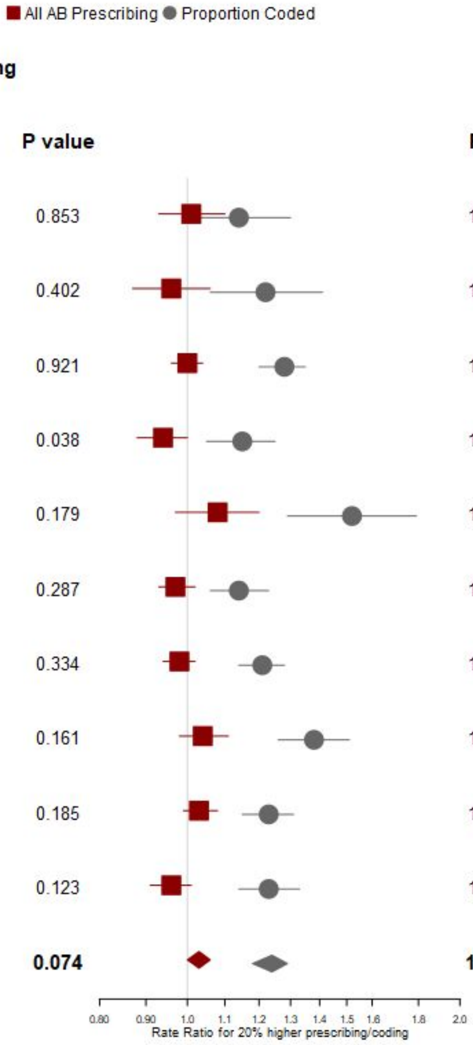

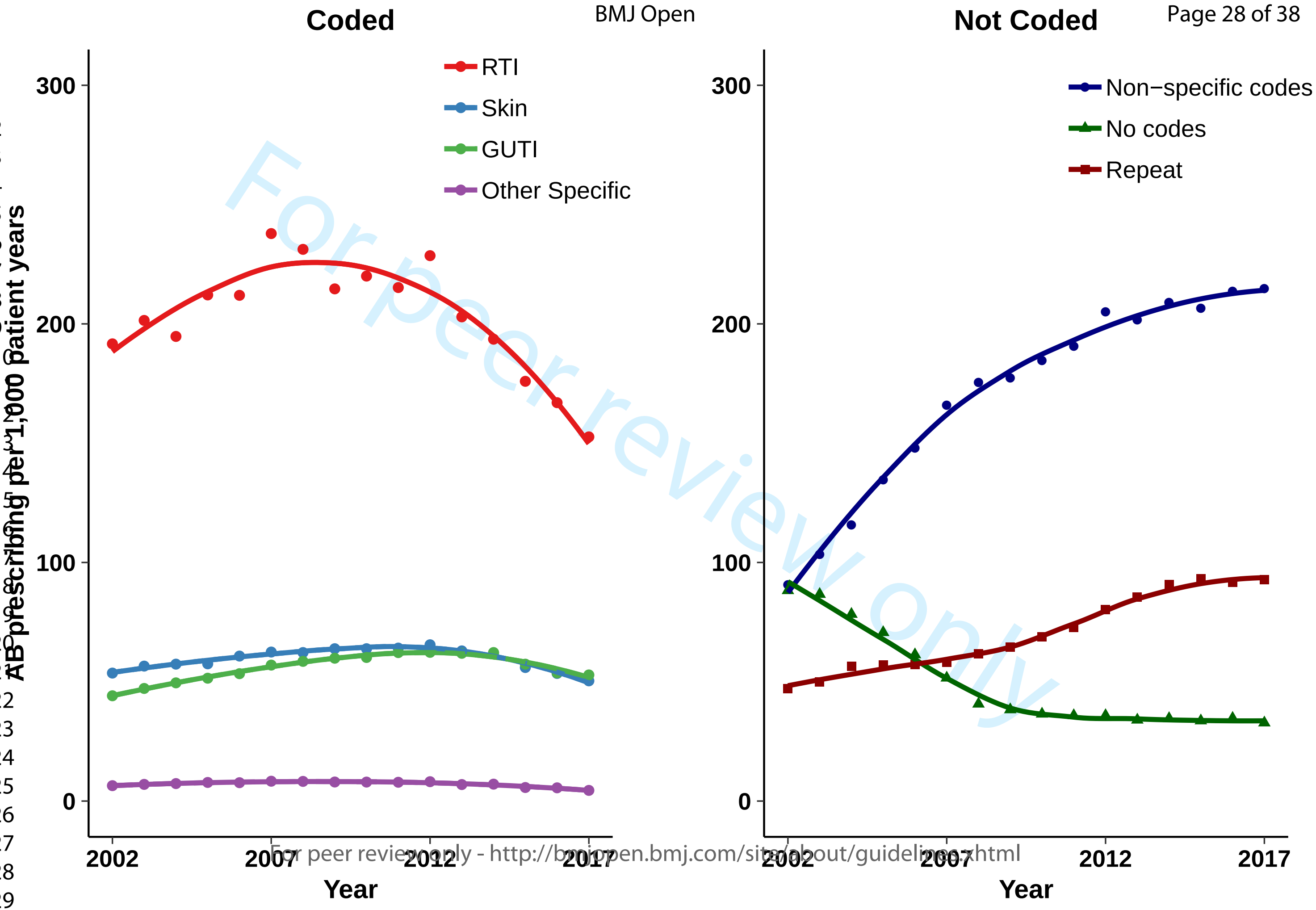

For peer review only

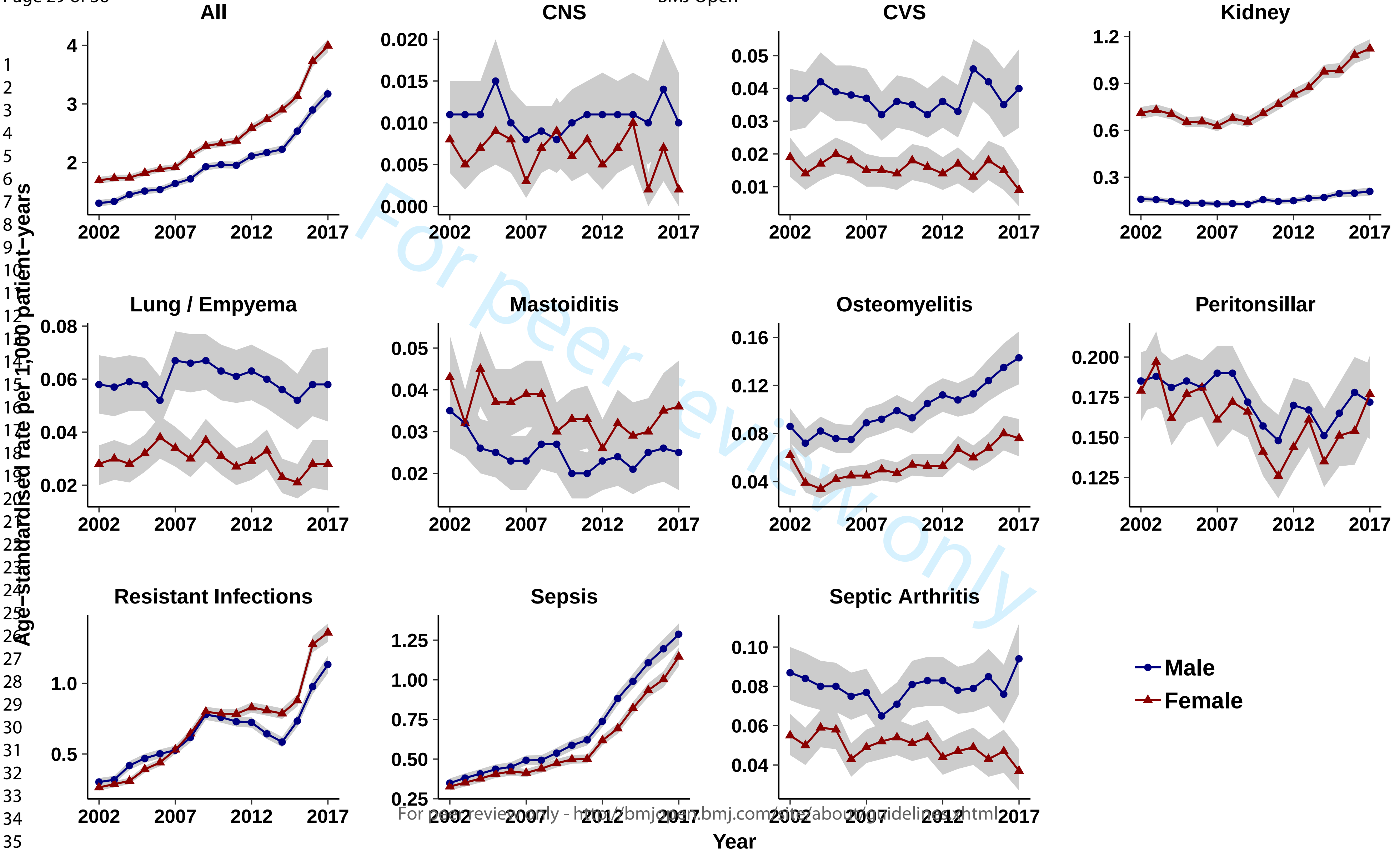

For peer review only

■ All AB Prescribing ● Proportion Coded

	All AB Prescribing				Medical Coding			
**Outcome**	**RR**	**LL**	**UL**	**P value**	**RR**	**LL**	**UL**	**P value**
CNS infection	1.01	0.93	1.10	0.853	1.14	1.01	1.30	0.039
CVS infection	0.96	0.87	1.06	0.402	1.22	1.06	1.41	0.005
Kidney infection	1.00	0.96	1.04	0.921	1.28	1.20	1.35	0.000
Lung abscess / Empyema	0.94	0.88	1.00	0.038	1.15	1.05	1.25	0.002
Mastoiditis	1.08	0.97	1.20	0.179	1.52	1.29	1.79	0.000
Osteomyelitis	0.97	0.93	1.02	0.287	1.14	1.06	1.23	0.001
Peritonsillar abscess	0.98	0.94	1.02	0.334	1.21	1.14	1.28	0.000
Resistant infection	1.04	0.98	1.11	0.161	1.38	1.26	1.51	0.000
Sepsis	1.03	0.99	1.08	0.185	1.23	1.15	1.31	0.000
Septic Arthritis	0.96	0.91	1.01	0.123	1.23	1.14	1.33	0.000
**Combined**	**1.03**	**1.00**	**1.06**	**0.074**	**1.24**	**1.18**	**1.29**	**0.000**

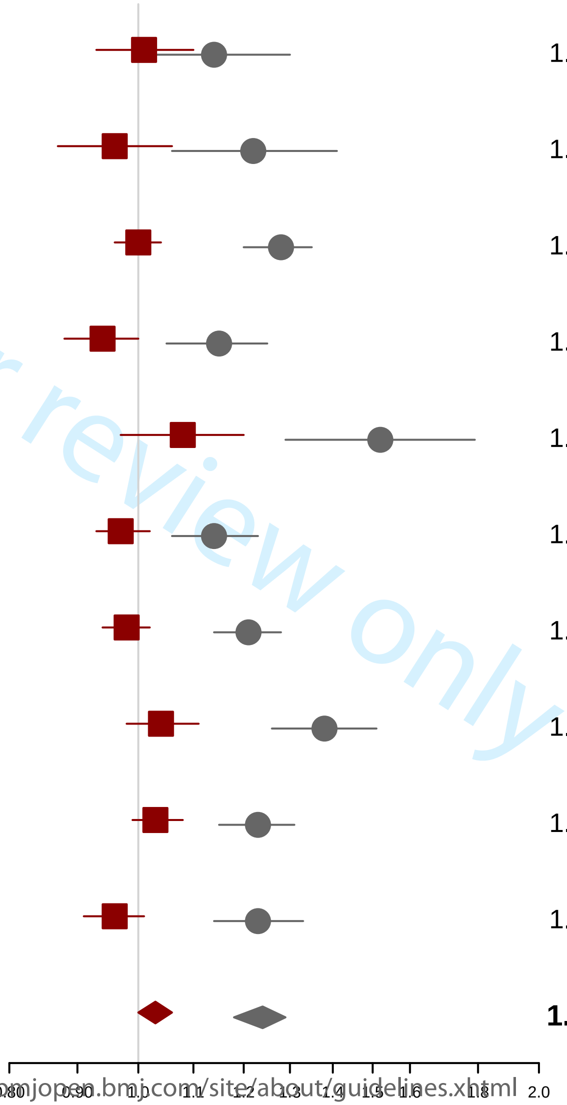

For peer review only

Gulliford et al. Supplementary Data

Supplementary Table 1: Outline of statistical models employed for analysis. (Please see text for further explanation).

**Model 1**	Antibiotic_count ~ year+year^2+year^3+age_group+gender+comorbidity+deprivation+region+offset=log(person-years)+random_intercept(family_practice), model=Poisson
**Model 2**	Serious_bacterial_infection_count ~ Family_Practice_AB_Prescribing_Estimate+Family_Practice_Consultations_Coded_Estimate+year+year^2+year^3+gender+ age_group+deprivation+region+offset=log(person-years)+random_intercept(family_practice), model=Poisson

For peer review only - http://bmjopen.bmj.com/site/about/guidelines.xhtml

Figure 1: Flowchart showing classification of antibiotic prescriptions 2002 to 2017. Figures are frequencies (percent of total number of antibiotic prescriptions).

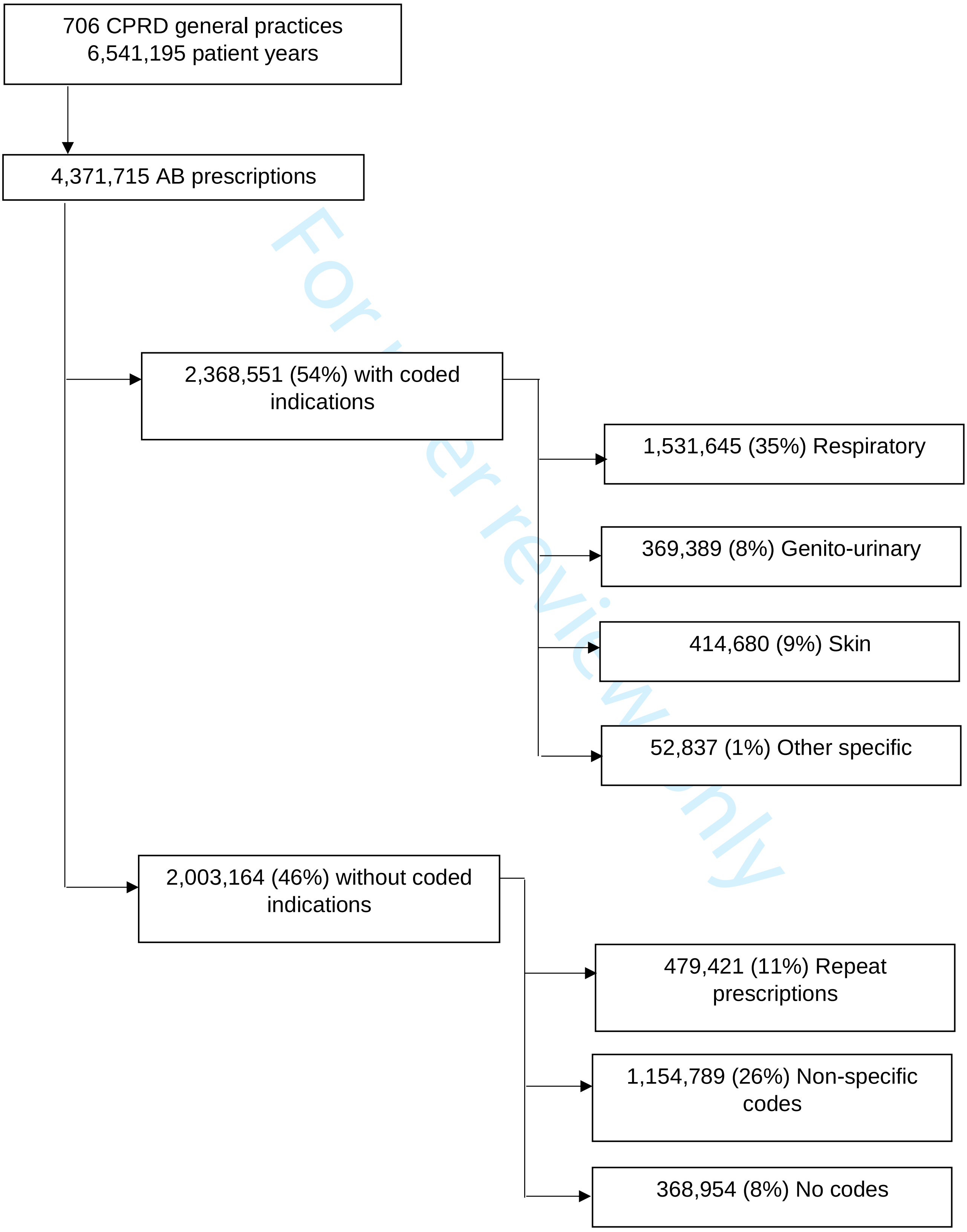

For peer review only - http://bmjopen.bmj.com/site/about/guidelines.xhtml

Gulliford et al. Supplementary Data

Supplementary Table 2: Characteristics of sample and antibiotic prescriptions by time period. Figures are frequencies (column percent).

	2002 to 2006	2007 to 2012	2013 to 2017
**Number of general practices**	652	672	589
**Number of patients contributing person time[a]**	548,558	576,985	439,627
**Number of person years**	2,253,436	2,768,176	1,519,582
Age 0 to 4	275,539	313,806	104,688
Age 5 to 14	371,352	611,610	393,224
Age 85+	169,709	216,966	111,606
Comorbidity present[a]	835,565	1,147,828	686,777
**Number of antibiotic prescriptions**	1,422,009	1,941,102	1,008,604
Acute AB prescriptions	1,289,615 (91)	1,739,666 (90)	863,013 (86)
for RTI	534,535 (38)	705,262 (36)	291,848 (29)
for GUTI	115,928 (8)	166,336 (9)	87,125 (9)
for skin infection	137,936 (10)	184,420 (10)	92,324 (9)
Other specific codes recorded	18,277 (1)	24,849 (1)	9,711 (1)
Non-specific codes recorded	290,472 (20)	537,110 (28)	327,207 (32)
No codes recorded	192,467 (14)	121,689 (6)	54,798 (5)
Repeat AB prescriptions	132,394 (9)	201,436 (10)	145,591 (15)

[a] figures were rounded to nearest whole number; [b]based on seasonal-influenza risk status

For peer review only - http://bmjopen.bmj.com/site/about/guidelines.xhtml

Gulliford et al. Supplementary Data

Supplementary Figure 2: Age-standardised antibiotic prescribing rates per 1,000 patient years for males and females from 2002 to 2017.

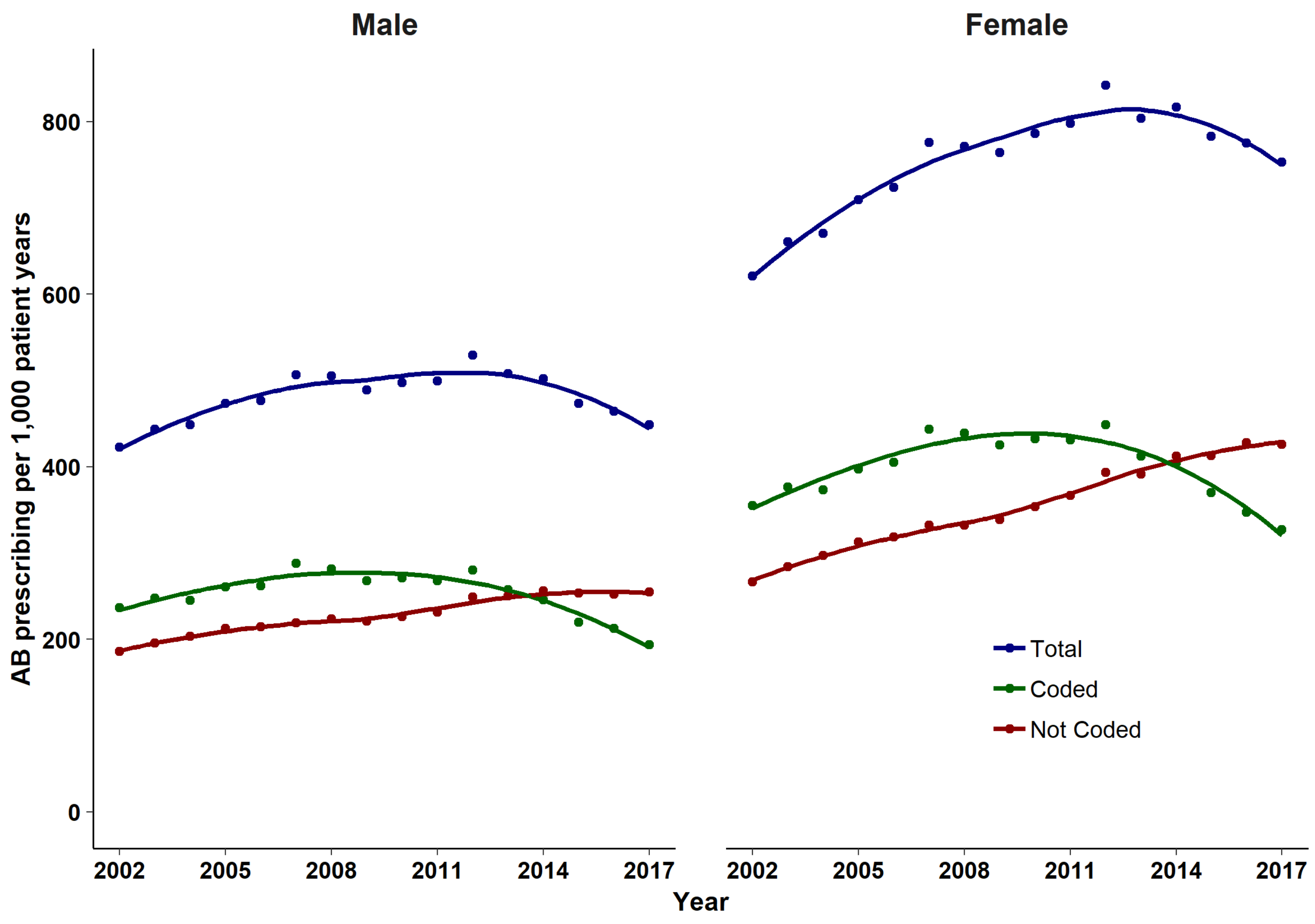

Gulliford et al. Supplementary Data

Supplementary Table 3: Distribution of serious bacterial infection events 2002 to 2017 by age-group and gender. Figures are frequencies.

	CNS infection	CVS infection	Kidney infection	Lung abscess / empyema	Mastoiditis	Osteomyelitis	Peritonsillar abscess	AB resistant infections	Sepsis	Septic arthritis
All	576	1,697	30,827	2,932	1,970	4,921	11,338	42,185	39,059	4,254
Male	352	1,144	4,997	1,903	814	3,055	6,021	18,312	18,999	2,496
Female	224	553	25,830	1,029	1,156	1,866	5,317	23,873	20,060	1,758
**Age-group (years)**										
0 to 4	11	20	198	138	178	138	73	576	469	147
5 to 9	17	18	386	106	153	118	232	409	334	104
10 to 14	17	17	474	60	111	167	465	308	244	93
15 to 24	47	42	6,140	106	167	152	3,428	1,528	970	129
25 to 34	38	92	5,523	149	203	160	2,621	2,444	1,474	243
35 to 44	65	146	5,176	294	280	392	2,483	3,089	2,164	392
45 to 54	115	189	4,519	438	270	635	1,079	4,001	3,345	555
55 to 64	105	274	3,725	561	255	865	553	5,045	5,385	678
65 to 74	90	407	2,562	525	210	937	285	8,252	7,817	775
75 to 84	58	365	1,548	423	109	924	94	9,469	9,646	727
85+	13	127	576	132	34	433	24	7,064	7,211	411

For peer review only

Gulliford et al. Supplementary Data

Supplementary Table 4: Age- and sex-standardised incidence rates per 1,000 patient-years for serious bacterial infections by quartile of antibiotic prescribing.

	General practice antibiotic prescribing[a]		General practice medical coding[a]	
**Group**	**Lowest fourth of general practices**	**Highest fourth of general practices**	**Lowest fourth of general practices**	**Highest fourth of general practices**
All	1.86 (1.83 to 1.88)	2.23 (2.20 to 2.25)	1.94 (1.91 to 1.96)	2.39 (2.37 to 2.42)
CNS Infection	0.008 (0.007 to 0.010)	0.009 (0.008 to 0.011)	0.008 (0.007 to 0.009)	0.010 (0.009 to 0.012)
CVS infection	0.024 (0.021 to 0.027)	0.026 (0.023 to 0.028)	0.026 (0.024 to 0.029)	0.027 (0.025 to 0.030)
Kidney Infection	0.40 (0.39 to 0.41)	0.49 (0.48 to 0.50)	0.37 (0.37 to 0.38)	0.55 (0.53 to 0.56)
Lung abscess / empyema	0.042 (0.039 to 0.045)	0.045 (0.042 to 0.049)	0.044 (0.041 to 0.047)	0.049 (0.046 to 0.053)
Mastoiditis	0.025 (0.022 to 0.027)	0.033 (0.030 to 0.036)	0.021 (0.019 to 0.023)	0.036 (0.033 to 0.039)
Osteomyelitis	0.071 (0.067 to 0.075)	0.073 (0.069 to 0.077)	0.071 (0.067 to 0.075)	0.081 (0.077 to 0.086)
Peritonsillar abscess	0.16 (0.15 to 0.17)	0.16 (0.16 to 0.17)	0.14 (0.14 to 0.15)	0.17 (0.17 to 0.18)
Resistant infections & C. difficile	0.50 (0.49 to 0.51)	0.68 (0.67 to 0.69)	0.63 (0.62 to 0.64)	0.73 (0.72 to 0.74)
Sepsis	0.57 (0.56 to 0.58)	0.65 (0.63 to 0.66)	0.56 (0.55 to 0.57)	0.67 (0.66 to 0.68)
Septic arthritis	0.064 (0.059 to 0.068)	0.064 (0.060 to 0.068)	0.057 (0.053 to 0.061)	0.068 (0.064 to 0.072)

[a]quartiles were estimated from a hierarchical regression model adjusting for age-group, gender, comorbidity, region, deprivation and year

For peer review only - http://bmjopen.bmj.com/site/about/guidelines.xhtml

Gulliford et al. Supplementary Data

Supplementary Figure 3: Forest plot showing the adjusted rate ratio for each type of serious bacterial infection for 20% higher total AB prescribing (red) or repeat AB prescribing (grey). Estimates were adjusted for each variable shown and gender, age-group, comorbidity, deprivation fifth, region, year (including quadratic and cubic terms).

■ Acute AB prescribing ● Repeat AB prescribing

	Acute Prescribing				Repeat Prescribing			
**Outcome**	**RR**	**LL**	**UL**	**P value**	**RR**	**LL**	**UL**	**P value**
CNS infection	1.01	0.93	1.09	0.812	0.99	0.96	1.02	0.593
CVS infection	0.94	0.86	1.03	0.186	1.02	0.99	1.06	0.225
Kidney infection	0.98	0.94	1.01	0.197	1.03	1.02	1.05	0.000
Lung abscess / Empyema	0.92	0.87	0.97	0.003	1.03	1.00	1.05	0.025
Mastoiditis	1.04	0.94	1.15	0.417	1.04	1.00	1.09	0.051
Osteomyelitis	0.96	0.91	1.00	0.055	1.03	1.01	1.05	0.008
Peritonsillar abscess	0.96	0.92	0.99	0.022	1.03	1.02	1.05	0.000
Resistant infection	1.04	0.98	1.10	0.203	1.00	0.98	1.03	0.752
Sepsis	1.03	0.99	1.07	0.186	1.00	0.98	1.02	0.972
Septic Arthritis	0.94	0.89	0.98	0.007	1.03	1.01	1.05	0.006
**Combined**	**1.02**	**0.99**	**1.05**	**0.227**	**1.01**	**1.00**	**1.02**	**0.054**

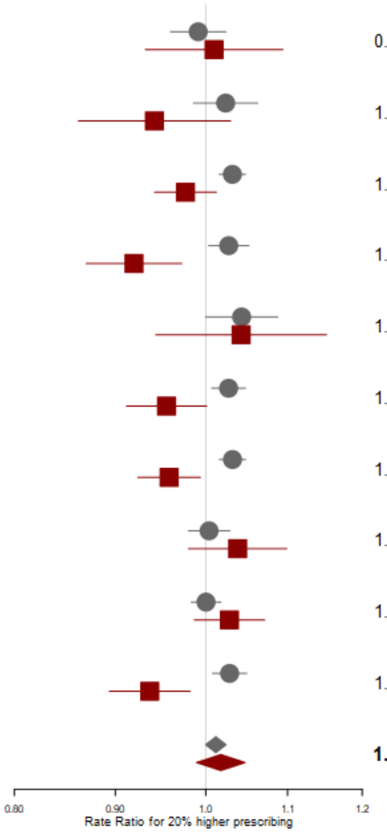

STROBE Statement—Checklist of items that should be included in reports of ***cohort studies***

	Item No	Recommendation	
**Title and abstract**	1	(*a*) Indicate the study's design with a commonly used term in the title or the abstract	P1
		(*b*) Provide in the abstract an informative and balanced summary of what was done and what was found	P3
**Introduction**			
Background/rationale	2	Explain the scientific background and rationale for the investigation being reported	P4-5
Objectives	3	State specific objectives, including any prespecified hypotheses	P5
**Methods**			
Study design	4	Present key elements of study design early in the paper	P6
Setting	5	Describe the setting, locations, and relevant dates, including periods of recruitment, exposure, follow-up, and data collection	P6
Participants	6	(*a*) Give the eligibility criteria, and the sources and methods of selection of participants. Describe methods of follow-up	P6
		(*b*) For matched studies, give matching criteria and number of exposed and unexposed	N/A
Variables	7	Clearly define all outcomes, exposures, predictors, potential confounders, and effect modifiers. Give diagnostic criteria, if applicable	P6-8
Data sources/ measurement	8*	For each variable of interest, give sources of data and details of methods of assessment (measurement). Describe comparability of assessment methods if there is more than one group	P6
Bias	9	Describe any efforts to address potential sources of bias	P8/9
Study size	10	Explain how the study size was arrived at	P6
Quantitative variables	11	Explain how quantitative variables were handled in the analyses. If applicable, describe which groupings were chosen and why	P6-8
Statistical methods	12	(*a*) Describe all statistical methods, including those used to control for confounding	P8/9
		(*b*) Describe any methods used to examine subgroups and interactions	P8/9
		(*c*) Explain how missing data were addressed	P8/9
		(*d*) If applicable, explain how loss to follow-up was addressed	N/A
		(*e*) Describe any sensitivity analyses	P12/13
**Results**			
Participants	13*	(a) Report numbers of individuals at each stage of study—eg numbers potentially eligible, examined for eligibility, confirmed eligible, included in the study, completing follow-up, and analysed	P10, ST2
		(b) Give reasons for non-participation at each stage	N/A
		(c) Consider use of a flow diagram	SF1
Descriptive data	14*	(a) Give characteristics of study participants (eg demographic, clinical, social) and information on exposures and potential confounders	ST2
		(b) Indicate number of participants with missing data for each variable of interest	SF1
		(c) Summarise follow-up time (eg, average and total amount)	P8, P10
Outcome data	15*	Report numbers of outcome events or summary measures over time	ST3
Main results	16	(*a*) Give unadjusted estimates and, if applicable, confounder-adjusted	Fig3

For peer review only

		estimates and their precision (eg, 95% confidence interval). Make clear which confounders were adjusted for and why they were included	P8/9, ST1
		(*b*) Report category boundaries when continuous variables were categorized	P7/8
		(*c*) If relevant, consider translating estimates of relative risk into absolute risk for a meaningful time period	P13
Other analyses	17	Report other analyses done—eg analyses of subgroups and interactions, and sensitivity analyses	P11
**Discussion**			
Key results	18	Summarise key results with reference to study objectives	P13
Limitations	19	Discuss limitations of the study, taking into account sources of potential bias or imprecision. Discuss both direction and magnitude of any potential bias	P14-15
Interpretation	20	Give a cautious overall interpretation of results considering objectives, limitations, multiplicity of analyses, results from similar studies, and other relevant evidence	P16
Generalisability	21	Discuss the generalisability (external validity) of the study results	P16
**Other information**			
Funding	22	Give the source of funding and the role of the funders for the present study and, if applicable, for the original study on which the present article is based	P17

*Give information separately for exposed and unexposed groups.

Note: An Explanation and Elaboration article discusses each checklist item and gives methodological background and published examples of transparent reporting. The STROBE checklist is best used in conjunction with this article (freely available on the Web sites of PLoS Medicine at http://www.plosmedicine.org/, Annals of Internal Medicine at http://www.annals.org/, and Epidemiology at http://www.epidem.com/). Information on the STROBE Initiative is available at http://www.strobe-statement.org.

For peer review only

BMJ Open

SERIOUS BACTERIAL INFECTIONS AND ANTIBIOTIC PRESCRIBING IN PRIMARY CARE. COHORT STUDY USING ELECTRONIC HEALTH RECORDS IN THE UK

Journal:	*BMJ Open*
Manuscript ID	bmjopen-2020-036975.R1
Article Type:	Original research
Date Submitted by the Author:	31-Jan-2020
Complete List of Authors:	Gulliford, Martin; King's College London, UK,<br>Sun, Xiaohui; King's College London, School of Population and Environmental Health Sciences<br>Charlton, Judith; King's College London, UK, Primary Care and Public Health Sciences<br>Winter, Joanne; King's College London, UK<br>Bunce, Catey; kings College london, Primary Care and Public Health Sciences<br>Boiko, Olga; King's College London, UK<br>Fox, Robin; Bicester Health Centre<br>Little, Paul; University of Southampton, Primary Care and Population Science;<br>Moore, Michael; University of Southampton Medical School, Primary Care Medical Group<br>Hay, Alastair; University of Bristol, Centre for Academic Primary Care<br>Ashworth, Mark; King's College London, UK, School of Population Health & Environmental Sciences
**Primary Subject Heading**:	Epidemiology
Secondary Subject Heading:	General practice / Family practice, Infectious diseases, Public health
Keywords:	PRIMARY CARE, Respiratory infections < THORACIC MEDICINE, Urinary tract infections < UROLOGY, Diagnostic microbiology < INFECTIOUS DISEASES

SCHOLARONE™ Manuscripts

I, the Submitting Author has the right to grant and does grant on behalf of all authors of the Work (as defined in the below author licence), an exclusive licence and/or a non-exclusive licence for contributions from authors who are: i) UK Crown employees; ii) where BMJ has agreed a CC-BY licence shall apply, and/or iii) in accordance with the terms applicable for US Federal Government officers or employees acting as part of their official duties; on a worldwide, perpetual, irrevocable, royalty-free basis to BMJ Publishing Group Ltd ("BMJ") its licensees and where the relevant Journal is co-owned by BMJ to the co-owners of the Journal, to publish the Work in this journal and any other BMJ products and to exploit all rights, as set out in our licence.

The Submitting Author accepts and understands that any supply made under these terms is made by BMJ to the Submitting Author unless you are acting as an employee on behalf of your employer or a postgraduate student of an affiliated institution which is paying any applicable article publishing charge ("APC") for Open Access articles. Where the Submitting Author wishes to make the Work available on an Open Access basis (and intends to pay the relevant APC), the terms of reuse of such Open Access shall be governed by a Creative Commons licence – details of these licences and which Creative Commons licence will apply to this Work are set out in our licence referred to above.

Other than as permitted in any relevant BMJ Author's Self Archiving Policies, I confirm this Work has not been accepted for publication elsewhere, is not being considered for publication elsewhere and does not duplicate material already published. I confirm all authors consent to publication of this Work and authorise the granting of this licence.

For peer review only

SERIOUS BACTERIAL INFECTIONS AND ANTIBIOTIC PRESCRIBING IN PRIMARY CARE. COHORT STUDY USING ELECTRONIC HEALTH RECORDS IN THE UK

Martin C Gulliford MA FRCP,[1,2]	Professor of Public Health
Xiaohui Sun MPH,[1]	PhD student
Judith Charlton MSc,[1]	Research Associate
Joanne R. Winter PhD,[1]	Research Associate
Catey Bunce PhD,[1,2]	Reader in Medical Statistics
Olga Boiko PhD,[1]	Research Associate
Robin Fox MB FRCGP,[3]	General Practitioner
Paul Little MD,[5]	Professor of Primary Care Research
Michael V. Moore BM FRCGP,[5]	Professor of Primary Health Care Research
Alastair D Hay MD,[4]	General Practitioner and Professor of Primary Care
Mark Ashworth DM[1]	Reader in General Practice

And SafeAB Research Group

[1]School of Population Health and Environmental Sciences, King's College London, Guy's Campus, King's College London, London SE1 1UL, UK;
[2]NIHR Biomedical Research Centre at Guy's and St Thomas' Hospitals London, Great Maze Pond, London SE1 9RT, UK;
[3]The Health Centre, Coker Close, Bicester, Oxfordshire, OX26 6AT, UK;
[4]Primary Care Research Group, University of Southampton, Aldermoor Health Centre, Aldermoor Close, Southampton, SO16 5ST, UK;
[5]Centre for Academic Primary Care, Bristol Medical School, Population Health Sciences, University of Bristol, 39 Whatley Rd, Bristol BS8 2PS, UK;

Correspondence: Martin Gulliford
martin.gulliford@kcl.ac.uk
Addison House, Guy's Campus,
King's College London, London SE1 1UL
Tel: 0207 848 6631
Fax: 0207 848 6620

Word count:

Text	3,596 words
Abstract	278 words
Tables	2
Figures	3

For peer review only - http://bmjopen.bmj.com/site/about/guidelines.xhtml

ABSTRACT

Objective: This study evaluated whether serious bacterial infections are more frequent at family practices with lower antibiotic prescribing rates.

Design: Cohort study.

Setting: 706 UK family practices in the Clinical Practice Research Datalink from 2002 to 2017.

Participants: 10.1 million registered patients with 69.3 million patient-years' follow-up.

Exposures: All antibiotic prescriptions, sub-groups of acute and repeat antibiotic prescriptions, and proportion of antibiotic prescriptions associated with specific-coded indications.

Main outcome measures: First episodes of serious bacterial infections. Poisson models were fitted adjusting for age-group, gender, comorbidity, deprivation, region and calendar year, with random intercepts representing family practice-specific estimates.

Results: The age-standardised antibiotic prescribing rate per 1,000 patient-years increased from 2002 (male 423; female 621) to 2012 (male 530; female 842) before declining to 2017 (male 449; female 753). The median family practice had an antibiotic prescribing rate of 648 per 1,000 patient-years with 95% range for different practices of 430 to 1,038 antibiotic prescriptions per 1,000 patient-years. Specific coded indications were recorded for 58% of antibiotic prescriptions at the median family practice, the 95% range at different family practices was from 10% to 75%. There were 139,759 first episodes of serious bacterial infection. After adjusting for covariates and the proportion of coded consultations, there was no evidence that serious bacterial infections were lower at family practices with higher total antibiotic prescribing. The adjusted rate ratio (RR) for 20% higher total antibiotic prescribing was 1.03, (95% confidence interval 1.00 to 1.06, P=0.074).

Conclusions: We did not find population-level evidence that family practices with lower total antibiotic prescribing might have more frequent occurrence of serious bacterial infections overall. Improving the recording of infection episodes has potential to inform better antimicrobial stewardship in primary care.

Key words: antibiotics; primary care; respiratory tract infections; peritonsillar abscess; mastoiditis.

[281 words]

For peer review only - http://bmjopen.bmj.com/site/about/guidelines.xhtml

For peer review only

Strengths and limitations of this study

- This cohort study included 10.1 million patients with 69.3 million patient-years of follow-up at 706 UK family practices from 2002 to 2017.
- The study included all antibiotic prescriptions and classified them according to the medical conditions recorded on the same date
- The study relied on medical conditions recorded by health care professionals in primary care
- Missing and misclassified information might result in bias, which might generally be towards a null finding
- The study aimed to evaluate associations at the general practice-level and the results do not exclude the possibility of association at the individual patient-level

For peer review only

For peer review only - http://bmjopen.bmj.com/site/about/guidelines.xhtml

INTRODUCTION

Antimicrobial resistance is a growing concern for health systems. The G20 health ministers noted that ‘drug-resistant [organisms] are to blame for 700,000 deaths worldwide each year, and this figure is predicted to rise to 10 million by 2050 if urgent action is not taken.’ (1) There are now intense efforts to reduce unnecessary use of antibiotics, especially in primary care where 80% of antibiotics are prescribed. These antimicrobial stewardship programmes have met with some success. In England, the total quantity of antibiotics prescribed in primary care declined by 13.2% in the 5 years between 2013 and 2017.(2, 3) Bacterial infections are still of public health importance with 1.7 million cases of sepsis and 270,000 deaths per year in the U.S.(4) Strategies to reduce inappropriate use of antibiotics must ensure that antibiotics can be used when they are needed.(5, 6)

It is possible that reducing antibiotic prescribing might be associated with greater risk of serious bacterial infections. Previous research investigated infection risk and antibiotic prescribing for respiratory illnesses.(3, 7) In a cohort study, Petersen et al.(8) found that antibiotic treatment reduced risks of mastoiditis after otitis media, peritonsillar abscess after sore throat, and pneumonia after respiratory infection. An analysis of electronic health records,(9) found that family practices that prescribed antibiotic more frequently to patients with self-limiting respiratory illnesses might have lower risk of pneumonia and peritonsillar abscess but there were no associations with risk of mastoiditis, empyema, meningitis, intracranial abscess or Lemierre’s syndrome. A cluster- randomised trial of an antimicrobial stewardship intervention for respiratory prescribing,(10) as well as an interrupted time series analysis found no clear evidence that antimicrobial stewardship policies might be associated with increased bacterial infections overall.(11) However, Gharbi et al.(12) found that apparent non-use of antibiotics for urinary infections might be associated with higher risk of sepsis.

For peer review only

It is important to extend these investigations to include antibiotic prescribing for all indications because the reasons for antibiotic prescribing may not always be well-documented, with up to half of antibiotic prescriptions in UK primary care not associated with any record of specific diagnostic medical codes.(3, 7) When analyses are restricted to antibiotic prescriptions for clearly recorded indications, the true extent of antibiotic prescribing may be under-estimated. It is also important to assess repeat antibiotic prescriptions which may be given for prevention of recurrent infections or treatment of serious or chronic infections.(3) The present study aimed to test the hypothesis that greater use of antibiotics for all indications might be associated with lower risk of serious bacterial infection. We also investigated whether patterns of medical coding were associated with the apparent occurrence of serious bacterial infection.

For peer review only

METHODS

Data source

We carried out a population-based cohort study in the UK Clinical Practice Research Datalink (CPRD) employing data for 2002 to 2017. The CPRD is one of the world's largest databases of primary care electronic health records, with participation of about 7% of UK family practices and with ongoing collection of anonymised data from 1990.(13) The high quality of CPRD data has been confirmed in many studies.(14) In order to estimate family practice-level prescribing metrics, we analysed a sample of CPRD data. This was because it was not feasible to analyse all antibiotic prescription for the whole of CPRD because the resulting dataset would have been too large for analysis. However, we ascertained serious bacterial infection events from the entire population of CPRD because these are generally rare events. The protocol for the study has been published. The protocol was approved by the CPRD Independent Scientific Advisory Committee (ISAC protocol 18-041R).

Selection of sample for antibiotic prescribing analysis

In order to analyse antibiotic prescribing, a sample was drawn from the CPRD denominator file for the October 2018 release of CPRD. A random sample of registered patients was drawn, stratifying by year between 2002 and 2017 and by family practice. In each year of study, a sample of 10 participants was taken for each gender and age group using five-year age groups up to a maximum of 104 years. Each sampled participant contributed data in multiple years of follow-up. There was a total sample of 671,830 individual participants, registered at a total of 706 family practices, who contributed person time between 2002 and 2017. The sampling design enabled estimation of all age-specific rates with similar precision, while age-standardisation provided weightings across age groups.

For peer review only - http://bmjopen.bmj.com/site/about/guidelines.xhtml

Main measures for antibiotic prescribing

For each participant in the antibiotic prescribing sample, we calculated the person-time at risk between the start and end of the patient's record. Person time was grouped by gender, age-group and comorbidity. Age groups were from 0 to 4, 5 to 9 and 10 to 14 and then 10-year age groups up to 85 years and over. Comorbidity was evaluated as either present or absent in each person-year using the 'seasonal flu at risk codes' which are used to identify individuals at higher risk of infection who may benefit from influenza vaccination,(15) as reported previously.(10) Seasonal flu at risk Read codes include medical diagnostic codes for overweight and obesity, coronary heart disease, chronic kidney disease, chronic liver disease, chronic neurological disease, chronic respiratory disease, diabetes mellitus and disorders of the immune system and drug product codes for asthma therapy, corticosteroid drugs and immunosuppressive drugs. Conditions were coded as present if they were ever diagnosed up to the end of the study year. Collectively, these provide a summary measure of potential susceptibility to infection complications.

Antibiotic prescriptions were evaluated using product codes for antibiotics listed in section 5.1 of the British National Formulary, excluding methenamine and drugs for tuberculosis, and leprosy. Different antibiotic classes and antibiotic doses were not considered further in this analysis. Multiple antibiotic prescription records on the same day were considered as a single antibiotic prescription. Medical codes recorded on the same date as the antibiotic prescription were used to classify the indication for prescription using categories of 'respiratory', 'genito-urinary', 'skin', and 'other specific' indications. All other codes were classified as 'non-specific' codes. (3) A prescriptions was classified as 'acute' if it was the first prescription in a sequence or 'repeat' prescription otherwise, as reported previously.(3) Antibiotic prescriptions that were not associated with medical codes and were not repeat prescriptions were classified as 'no codes recorded'.

For peer review only - http://bmjopen.bmj.com/site/about/guidelines.xhtml

Serious bacterial infections

Incident cases of serious bacterial infection were evaluated in the January 2019 release of CPRD for the years 2002 to 2017 with the CPRD denominator providing the person time at risk. CPRD records include details of consultations by general practice staff, as well as coded records of referrals to hospital or discharge letters from hospitals. The mean duration of follow-up was 6.9 years. Serious bacterial infections were selected for study from review of the International Classification of Diseases 10th revision,(16) the Read code classification(17) and through discussion with the research team. The final list of conditions is summarised in Table 1 and included: bacterial infections of the central nervous system (CNS); bacterial infections of the cardiovascular system (CVS); kidney infections; lung abscess and empyema; mastoiditis; osteomyelitis; peritonsillar abscess; resistant infections and C. difficile; sepsis and septic arthritis. Incident events were first records for each type of serious bacterial infection in a patient more than 12 months after the start of the patient record. However, a single patient might have first episodes of more than one type of bacterial infection. Possible recurrent events in the same patient were not evaluated further because, in electronic health records, it may not be possible to distinguish new occurrences from reference to ongoing or previous problems.

Statistical analysis

The analysis was in two stages. First, we estimated family practice-specific estimates for antibiotic prescribing; secondly, we evaluated whether these estimates were associated with the risk of serious bacterial infection. In the first stage of the analysis, we analysed antibiotic prescribing in primary care between 2002 and 2017 (Supplementary Table 1: Model 1). A hierarchical Poisson model was fitted using the ‘hglm’ package in the R program,(18) with counts of antibiotic prescriptions as the outcome and the log of person time as the offset. Estimates were adjusted for the fixed effects of gender, age-group, fifth of deprivation at family practice-level, comorbidity, and region in the UK. Calendar year was included as a

For peer review only - http://bmjopen.bmj.com/site/about/guidelines.xhtml

continuous predictor together with quadratic and cubic terms to allow for non-linear trends. Random intercepts were estimated for each family practice and each estimate represented the adjusted log relative rate for antibiotic prescribing at that practice compared with the overall mean. The proportion of antibiotic prescriptions that were associated with specific medical codes was analysed in a similar framework with coded prescriptions as the outcome and the log of antibiotic prescriptions as the offset.

In the second stage of analysis, serious bacterial infections were analysed as the outcome (Supplementary Table 1: Model 2). The antibiotic prescribing level for each family practice was included as a predictor using the family practice-specific estimates from Model 1. These estimates initially had a mean of zero and standard deviation of 0.19, consistent with an adjusted relative rate of antibiotic prescribing of 1.21 for a family practice with prescribing one standard deviation above the mean. Estimates were therefore standardised to give the change in serious bacterial infection for a 20% relative increase in antibiotic prescribing rate at a practice, because this represents a change of approximately one standard deviation. A 20% change generally represents a substantial change in antibiotic prescribing. We also estimated the change in serious bacterial infection for a 20% relative increase in proportion of antibiotic prescriptions with specific medical codes recorded at a family practice. Models were adjusted for age-group, gender, region, deprivation fifth, calendar year (including quadratic and cubic terms for the latter), with log of person-time as offset. The results were visualised using forest plots.(19)

For peer review only

RESULTS

There were 706 family practices included in the analysis, with 10.1 million registered patients and 69.3 million patient years of follow-up. In the sub-sample analysed for antibiotic prescribing, there were 706 family practices with 6,541,195 person-years of follow-up (Supplementary Figure 1 and Supplementary Table 2). There was a total of 4,371,715 antibiotic prescriptions between 2002 and 2017. This included 2,368,551 (54%) with coded indications including 1,531,645 (35%) associated with respiratory infections, 369,389 (8%) with genitourinary infections, 414,680 (10%) with skin infections and 52,837 (1%) with other specific indications. There were 2,003,164 (46%) of antibiotic prescriptions without specific coded indications consisting of 479,421 (11%) repeat prescriptions, 1,154,789 (26%) with non-specific medical codes recorded and 368,954 (8%) with no medical codes recorded.

Supplementary Figure 2 shows changes over time in age-standardised antibiotic prescribing rates per 1,000 patient years for coded and not coded indications. During the initial period of the study from 2002 to 2012, the age-standardised total antibiotic prescribing rate per 1,000 patient years increased from 2002 (male 423; female 621) to 2012 (male 530; female 842) before declining to 2017 (male 449; female 753). The recent decrease in total antibiotic prescribing was accompanied by a decline in antibiotic prescribing for coded indications, but antibiotic prescriptions that were not associated with specific coded indications continued to increase. There was evidence of a decline in antibiotic prescribing for respiratory illness from 2008 onwards (Figure 1) and after 2012 there was evidence of decreasing prescribing for genito-urinary and skin infections, as well as other specific indications. Throughout the period from 2002 to 2017, antibiotic prescriptions associated with non-specific codes increased as did repeat prescriptions. Antibiotic prescriptions that were not associated with medical codes declined initially but then remained constant (Figure 1).

For peer review only - http://bmjopen.bmj.com/site/about/guidelines.xhtml

Table 2 summarises variation in antibiotic prescribing metrics between family practices in the sample. The 95% range for family practice-specific antibiotic prescribing rates was from 430 to 1,038 antibiotic prescriptions per 1,000 person-years, with a median of 648 antibiotic prescriptions per 1,000 patient years. The 95% range for the proportion of repeat prescriptions was from 3% to 24%. The 95% range for the proportion of antibiotic prescriptions with specific coded indications recorded ranged from 10% to 75%.

There were 139,759 first episodes of serious bacterial infections (Supplementary Table 3). Figure 2 shows trends in the age-standardised incidence of serious bacterial infections from 2002 to 2017. The total incidence of serious bacterial infections increased during the period. This increase was largely accounted for by increases in sepsis, antibiotic resistant and C. difficile infections, kidney infections and osteomyelitis. The remaining conditions showed either stable incidence or slight declines. Supplementary Table 4 presents age- and sex-standardised incidence rates per 1,000 patient-years for serious bacterial infections for the highest and lowest fourths of antibiotic prescribing. There was no evidence that serious bacterial infections might be more frequent at family practices in the lowest fourth of antibiotic prescribing. In general, age- and sex-standardised incidence rates tended to be highest at family practices that were higher prescribers of antibiotics. Supplementary Table 4 also compares the incidence of serious bacterial infection for the lowest and highest fourths of medical coding. In the lowest quartile of practices a median of 38% antibiotic prescriptions were coded, compared with 70% for practices in the highest quartile. Family practices in the highest fourth of medical coding had an incidence of serious bacterial infection of 2.39 per 1,000 patient years (95% confidence interval 2.37 to 2.42) compared with 1.94 (1.91 to 1.96) in the lowest fourth of medical coding.

For peer review only

Figure 3 presents a forest plot for the association of each serious bacterial infection with 20% higher total antibiotic prescribing at a family practice. The combined estimate revealed that there was no evidence that higher total antibiotic prescribing was associated with lower incidence of serious bacterial infections (adjusted rate ratio 1.03, 95% confidence interval 1.00 to 1.06, P=0.074). When the 10 classes of serious bacterial infection were considered individually, there was no evidence that higher antibiotic prescribing might be associated with a lower incidence of infections. However, there was weak evidence of that lung abscess and empyema (RR 0.94, 0.88 to 1.00, P=0.038) might be lower at higher prescribing family practices. There was strong evidence that the recorded incidence of serious bacterial infections was associated with the coding of specific indications for a antibiotic prescriptions (adjusted rate ratio for a 20% increase in coding proportion 1.24, 1.18 to 1.29, P<0.001). This association held for each of the 10 classes of serious bacterial infections considered individually.

We conducted a sensitivity analysis by excluding repeat prescriptions that might not have been for acute infection episodes. There was no evidence that higher acute (non-repeat) antibiotic prescribing was associated with serious bacterial infections overall (RR 1.02, 0.99 to 1.05, P=0.227). (Supplementary Figure 3) There was evidence that higher acute antibiotic prescribing might be associated with lower incidence of lung abscess and empyema and septic arthritis. Osteomyelitis and peritonsillar abscess were not judged to be associated with acute antibiotic prescribing after controlling the false discovery rate. There was weak evidence that higher repeat antibiotic prescribing might be associated with higher incidence of serious bacterial infections overall (RR 1.01, 1.00 to 1.02, P=0.054) with evidence of this association for kidney infections, osteomyelitis, peritonsillar abscess and septic arthritis considered separately.

For peer review only - http://bmjopen.bmj.com/site/about/guidelines.xhtml

DISCUSSION

Principal findings

This study found that antibiotic prescribing increased from 2002 to 2012 but declined subsequently with changes over time being of larger magnitude for women than men. The incidence of serious bacterial infections in men and women rose steadily between 2002 and 2017, particularly for sepsis (men and women), osteomyelitis (mainly in men), and kidney infections (mainly in women). The research aimed to test the hypothesis that family practices with lower utilisation of antibiotics might have greater risk of serious bacterial infections. We evaluated the incidence of serious bacterial infections including 10 groups of infections that affect different systems of the body as well as sepsis (including septicaemia). We did not find evidence that family practices that prescribe antibiotics less frequently might have a higher incidence of serious bacterial infections. We found evidence that each type of serious bacterial infection was recorded more frequently at family practices that record diagnostic codes for a high proportion of antibiotic prescriptions suggesting that variation in the incidence of serious bacterial infection among family practices may be partly an artefact of data-recording. Measures are needed to improve the recording of infection episodes in primary care both when antibiotics are prescribed and when they are not. Repeat prescriptions account for a significant proportion of uncoded prescriptions (3) and repeat prescriptions might be indicated for prolonged or serious infections. Certain conditions may be associated with a higher rate of repeat antibiotic prescribing if there is initial treatment failure. For example, surgical intervention may eventually be required for treatment empyema, osteomyelitis or infective endocarditis. We conducted analyses after excluding repeat prescriptions and these analyses raised the possibility that family practices with lower acute (non-repeat) antibiotic prescribing might have higher incidence of lung abscess and empyema and septic arthritis. However, these analyses were not pre-planned, should be considered as hypothesis-generating and requiring confirmation in future studies. The incidence of these two conditions is less than one per 10,000 patients per year, and a

For peer review only - http://bmjopen.bmj.com/site/about/guidelines.xhtml

relative rate of 0.9 for a 20% increase in prescribing implies that at most one additional case might arise every 10 years from a 20% reduction in prescribing at a family practice with 10,000 registered patients.

Strengths and weaknesses of the study

The study drew on data for a large population comprising data for about 7% of the UK general population. In view of sample size constraints, antibiotic utilisation was estimated through analysis of data for a sample of patients, using hierarchical (multilevel) regression models to obtain family practice-specific antibiotic prescribing estimates. This contrasts with our previous study in which age- and sex-standardised rates were calculated from the data for each practice.(9) Use of a regression modelling approach enabled us to make optimal use of the data, as well as adjusting for covariates that are associated with variations in antibiotic prescribing (20) including comorbidity, deprivation, region and calendar year, in addition to age and sex.(21) Consistent with previous studies,(3, 7) we observed that nearly half of antibiotic prescriptions were not associated with specific coded indications. This suggests that total antibiotic prescribing is the most appropriate exposure measure for consideration, because indication-specific antibiotic prescribing may be associated with considerable misclassification. Serious bacterial infections were identified from medical diagnostic codes recorded into primary care electronic health records, which include general practice records of consultations, hospital referrals and discharges. Many studies have shown that these records have a high predictive value for a range of diagnoses, (14) but relying on a single data source can lead to under-estimation of the total number of events.(22) CPRD records are linked to hospital episode statistics (HES) but only for a subset of general practices in England, leading to a reduced sample size. Further research incorporating HES data is now underway and will be reported separately. There may be changes over time in the use of diagnostic categories, which might in part account for increasing diagnoses of ‘sepsis’. A study of U.S. hospitals’ data found that there was a 706%

For peer review only - http://bmjopen.bmj.com/site/about/guidelines.xhtml

increase in sepsis between 2003 and 2012, without any corresponding increase in positive blood cultures.(23) There was also an apparent increase in resistant infections but this might also be due in part to data recording changes and growing awareness of the problem of antimicrobial resistance, as well as true increases in resistant infections. An interrupted time series analysis,(11) offers an alternative approach to analysis but this might be susceptible to changes over time in unmeasured confounders such as code selection. The results of our study draw attention to the problem of poor coding quality in the context of infection management in primary care. Evidence from other studies suggests that missing values are typically missing not at random and the act of data recording may introduce a form of confounding by indication that may bias results.(24) In order to allow for this, we explicitly evaluated the extent to which differences in data recording between practices might account for variations in the incidence of serious bacterial infections. It is likely that misclassification of exposure and outcome variables, from incomplete data recording, might lead to under-estimation of associations, though the direction of bias cannot always be anticipated.(25) We adjusted for a summary measure of comorbidity. Our analyses do not exclude the possibility that there may be vulnerable sub-groups of patients, such as those with immunosuppression, who may be at increased risk if antibiotics are withheld.

Comparison with other studies

The trends in total antibiotic utilisation reported here are consistent with national trends based on aggregate data.(2) Neilly et al.(26) found that increasing prescription volumes in the period up to 2013 could be accounted for by increasing dose and duration of prescriptions but we found evidence of increased antibiotic prescribing based on numbers of prescriptions alone. Consistent with our findings, Balinskaite et al.(11) reported increasing rates of infection in English primary care and hospital admissions data from 2010 to 2017. Their time series analysis suggested that antimicrobial stewardship intervention in 2015 had no impact on bacterial infections overall but there was some evidence for increasing hospital

For peer review only - http://bmjopen.bmj.com/site/about/guidelines.xhtml

admissions for quinsy, decreasing hospital admissions for pyelonephritis and decreasing GP consultation rates for empyema. In a previous study, we found that peritonsillar abscess and pneumonia might be more frequent when family practices prescribe antibiotics less frequently for respiratory tract infections.(9) We did not include pneumonia in this study because we found that syndromes of 'chest infection' and 'pneumonia' may be difficult to distinguish in primary care records with evidence of code shifting between the two categories.(27) In the present study, the incidence of peritonsillar abscess was not associated with total antibiotic prescribing. Randomised trials suggest that antibiotics protect against peritonsillar abscess (28) so it is plausible that this condition might be associated with respiratory antibiotic prescribing but not total antibiotic prescribing.

Patient and public involvement

The protocol and results of the study were discussed at meetings with patients. Patients commented on the recent declining trend in antibiotic prescribing. They noted that avoiding antibiotics requires trade-offs between the limited benefits from antibiotic treatment, the side effects of antibiotic use, and the potential from longer-term problems from the increase in antimicrobial resistance. Patients considered that risks of serious bacterial infections were generally low at the present time. There is a need to communicate these results to patients and prescribers so that both groups can be aware of the wider contextual issue of antimicrobial resistance to inform antibiotic prescribing decisions.

Main conclusions

Family practices that reduce the amount of antibiotics prescribed do not risk any increase in serious bacterial infections overall. This finding does not exclude the possibility that serious bacterial infection may be associated with antibiotic prescribing patterns at individual patient-level. Consequently, reducing antibiotic utilisation in primary care will require a detailed

For peer review only - http://bmjopen.bmj.com/site/about/guidelines.xhtml

understanding of when antibiotics prescriptions are required and when they are not and increasing the quality of data recording with respect to antibiotic use should be a high priority. This study focused on population-level associations at the level of family practice. Future research should evaluate the associations at the level of the individual patient and the individual family practice consultation. This might provide primary care professionals and patients with objective evidence concerning levels risk that can inform decisions to prescribe or not prescribe antibiotics.

For peer review only

Acknowledgement

The SafeABStudy Group also includes Dr Caroline Burgess, Dr Vasa Curcin and Dr James Shearer.

Data sources

The study is based in part on data from the Clinical Practice Research Datalink obtained under license from the UK Medicines and Healthcare products Regulatory Agency. However, the interpretation and conclusions contained in this report are those of the authors alone.

Data sharing

Requests for access to data from the study should be addressed to martin.gulliford@kcl.ac.uk. The study protocol has been published. All proposals requesting data access will need to specify planned uses with approval of the study team and CPRD before data release.

Funding

The study is funded by the National Institute for Health Research (NIHR) Health Services and Delivery Programme (16/116/46). MG was supported by the NIHR Biomedical Research Centre at Guy's and St Thomas' Hospitals. The views expressed are those of the authors and not necessarily those of the NHS, the NIHR, or the Department of Health. The funder of the study had no role in study design, data collection, data analysis, data interpretation, or writing of the report. The authors had full access to all the data in the study and all authors shared final responsibility for the decision to submit for publication.

Conflict of Interest

The authors have no conflicts of interest.

Author Contributions

MG wrote the study protocol with advice from CB, RF, MA, PL, MM and AH; XS developed and piloted code sets and analyses for antibiotic prescribing; RF, PL, MM, AH and MA reviewed case definitions; JC programmed analyses and JW advised; MG completed data analyses and drafted the paper with advice from CB, RF, PL, MM, AH and MA; OB coordinated PPI input. All authors reviewed and contributed to the final draft. MG is guarantor.

For peer review only

REFERENCES

1. G20 Information Centre. *Declaration: G20 Meeting of Health Ministers 2018* [Available from: http://www.g20.utoronto.ca/2018/2018-10-04-health.html accessed 11th October 2019]

2. Public Health England. *English Surveillance Programme for Antimicrobial Utilisation and Resistance (ESPAUR) Report 2017*. London: Public Health England, 2017. [Available from https://assets.publishing.service.gov.uk/government/uploads/system/uploads/attachment_data/file/759975/ESPAUR_2018_report.pdf accessed 11th October 2019]

3. Sun X, Gulliford MC. Reducing antibiotic prescribing in primary care in England from 2014 to 2017: population-based cohort study. *BMJ open*. 2019;**9** (7):e023989.

4. Centers for Disease Control and Prevention. *Sepsis: Data and Reports.* Atlanta, GA: Centers for Disease Control and Prevention, 2019: Source: https://www.cdc.gov/sepsis/datareports/index.html accessed 28th October 2019.

5. Laxminarayan R, Matsoso P, Pant S, Brower C, Røttingen J-A, Klugman K, et al. Access to effective antimicrobials: a worldwide challenge. *The Lancet.* 2016; **387**(10014):168-75.

6. NHS England. *Quality Premium: 2016/17 Guidance for CCGs.* Leeds: NHS England, 2016.

7. Dolk FCK, Pouwels KB, Smith DRM, Robotham JV, Smieszek T. Antibiotics in primary care in England: which antibiotics are prescribed and for which conditions? *J Antimicrobial Chemother*. 2018;**73** (suppl_2):ii2-ii10.

8. Petersen I, Johnson AM, Islam A, Duckworth G, Livermore DM, Hayward AC. Protective effect of antibiotics against serious complications of common respiratory tract

For peer review only - http://bmjopen.bmj.com/site/about/guidelines.xhtml

infections: retrospective cohort study with the UK General Practice Research Database. *BMJ*. 2007; **335**: 982.

9. Gulliford MC, Moore MV, Little P, Hay AD, Fox R, Prevost AT, et al. Safety of reduced antibiotic prescribing for self limiting respiratory tract infections in primary care: cohort study using electronic health records. *BMJ* 2016;**354**:i3410.

10. Gulliford MC, Prevost AT, Charlton J, Juszczyk D, Soames J, McDermott L, et al. Effectiveness and safety of electronically delivered prescribing feedback and decision support on antibiotic use for respiratory illness in primary care: REDUCE cluster randomised trial. *BMJ*. 2019;**364**:l236.

11. Balinskaite V, Aylin P, Johnson AP, Holmes A. The Impact of a National Antimicrobial Stewardship Program on Antibiotic Prescribing in Primary Care: An Interrupted Time Series Analysis. *Clin Infect Dis.* 2019; **69** (2):233-242. doi: 10.1093/cid/ciy904.

12. Gharbi M, Drysdale JH, Lishman H, Goudie R, Molokhia M, Johnson AP, et al. Antibiotic management of urinary tract infection in elderly patients in primary care and its association with bloodstream infections and all cause mortality: population based cohort study. *BMJ.* 2019;**364**:l525.

13. Herrett E, Gallagher AM, Bhaskaran K, Forbes H, Mathur R, van Staa T, et al. Data Resource Profile: Clinical Practice Research Datalink (CPRD). *Int J Epidemiol*. 2015;**44** (3)**:** 827-36.

14. Herrett E, Thomas SL, Schoonen WM, Smeeth L, Hall AJ. Validation and validity of diagnoses in the General Practice Research Database: a systematic review. *Br J Clin Pharmacol.* 2010;**69** (1):4-14.

15. NHS Employers. *Seasonal flu at risk Read Codes 2015-2016*. Leeds: NHS Employers, 2016.

For peer review only - http://bmjopen.bmj.com/site/about/guidelines.xhtml

16. World Health Organization. *International Statistical Classification of Diseases and Related Health Problems 10th Revision 2010* [Available from: http://apps.who.int/classifications/icd10/browse/2010/en accessed 11th October 2019.

17. NHS Digital. *Clinical terms (Read codes). Summarised product description*. Leeds: NHS Digital, 2018. Available at https://isd.digital.nhs.uk/trud3/user/guest/group/0/pack/9/subpack/19/releases accessed 11th October 2019.

18. Lee Y, Ronnegard L, Noh M. *Data analysis using hierarchical generalized linear models with R.* Boca Raton, FL: CRC Press; 2017.

19. Gordon M, Lumley T. *Advanced Forest Plot Using 'grid' Graphics.* Vienna: The Comprehensive R Archive Network, 2016. Source: https://cran.r-project.org/web/packages/forestplot/forestplot.pdf. accessed 11th October 2019.

20. Pouwels KB, Dolk FCK, Smith DRM, Smieszek T, Robotham JV. Explaining variation in antibiotic prescribing between general practices in the UK. *J Antimicrobial Chemother* 2018;**73** (suppl_2):ii27-ii35.

21. Goldstein H, Spiegelhalter DJ. League Tables and Their Limitations: Statistical Issues in Comparisons of Institutional Performance. *J Royal Statistical Society, A*. 1996;**159** (3) :385-443.

22. Herrett E, Shah AD, Boggon R, Denaxas S, Smeeth L, van Staa T, et al. Completeness and diagnostic validity of recording acute myocardial infarction events in primary care, hospital care, disease registry, and national mortality records: cohort study. *BMJ.* 2013;**346**:f2350.

For peer review only - http://bmjopen.bmj.com/site/about/guidelines.xhtml

23. Rhee C, Murphy MV, Li L, Platt R, Klompas M. Comparison of trends in sepsis incidence and coding using administrative claims versus objective clinical data. *Clin infectious dis* 2015;**60**(1)**:**88-95.

24. Agniel D, Kohane IS, Weber GM. Biases in electronic health record data due to processes within the healthcare system: retrospective observational study. *BMJ*. 2018;**361:** k1479.

25. Greenland S, Robins JM. Confounding and misclassification. *Am J Epidemiol.* 1985;**122** (3):495-506.

26. Neilly MDJ, Guthrie B, Hernandez Santiago V, Vadiveloo T, Donnan PT, Marwick CA. Has primary care antimicrobial use really been increasing? Comparison of changes in different prescribing measures for a complete geographic population 1995–2014. *J Antimicrobial Chemother*. 2017;**72** (10)**:**2921-30.

27. Sun X, Douiri A, Gulliford M. Pneumonia incidence trends in UK primary care from 2002 to 2017: population-based cohort study. *Epidemiol Infect.* 2019;**147**:e263. doi: 10.1017/S0950268819001559.

28. Spinks A, Glasziou PP, Del Mar CB. Antibiotics for sore throat. *Cochrane database syst rev*. 2013;**11**:CD000023.

For peer review only - http://bmjopen.bmj.com/site/about/guidelines.xhtml

Table 1: Groups of serious bacterial infections including numbers of medical codes and five most frequently recorded conditions. Figures are frequencies.

Group	Number of codes	Number of first events	Five most frequent conditions (number of first events 2002 to 2017)
CNS Infection	30	576	Epidural abscess (117), cerebral abscess (112), brain abscess (79), intraspinal abscess (49), drainage of abscess of subdural space (44)
CVS infection	24	1,697	Acute and subacute endocarditis (594), bacterial endocarditis (276), Subacute bacterial endocarditis (270), acute endocarditis NOS (166), acute bacterial endocarditis (114)
Kidney Infection	22	30,827	Acute pyelonephritis (19.284), pyelonephritis unspecified (7,115), infections of kidney (1,670), acute pyelitis (1,008), pyelitis unspecified (745)
Lung abscess / empyema	24	2,932	Empyema (2,314), abscess of lung (149), abscess of lung and mediastinum (139), thorax abscess NOS (68), pleural empyema (56)
Mastoiditis	10	1,970	Mastoiditis and related conditions (1,293), mastoiditis NOS (487), acute mastoiditis (146), acute mastoiditis NOS (31), abscess of mastoid (27)
Osteomyelitis	65	4,921	Acute osteomyelitis (3,297), unspecified osteomyelitis (678), unspecified osteomyelitis of unspecified site (284), osteomyelitis jaw (78), unspecified osteomyelitis NOS (75)
Peritonsillar abscess	6	11,338	Quinsy (8,611), peritonsillar abscess – quinsy (1,748), O/E quinsy present (654), drainage of peritonsillar abscess (232), drainage of quinsy (226),
Resistant infections & C. difficile	31	42,185	Clostridium difficile toxin detection (20,175), methicillin resistant staphylococcus aureus positive (9,914), Clostridium difficile infection (6,397), methicillin resistant staphylococcus aureus (4,303), methicillin resistant staphylococcus aureus carrier (1,017)
Sepsis	100	39,059	Sepsis (23,149), septicaemia (6,204), urosepsis (4,646), biliary sepsis (1,233), Clostridium infection (576)
Septic arthritis	41	4,254	Septic arthritis (3,649), Pyogenic arthritis (184), Arthropathy associated with infections (172), Knee pyogenic arthritis (52), Staphylococcal arthritis and polyarthritis (39)

For peer review only - http://bmjopen.bmj.com/site/about/guidelines.xhtml

Table 2: Variation in antibiotic prescribing between family practices. Figures represent the centiles of the distribution of family practice-specific values.

	Centiles of family practices				
**Measure**	**2.5th**	**25th**	**Median**	**75th**	**97.5th**
AB prescribing rate per 1,000 patient-years	430	563	648	748	1,038
Acute prescriptions (% of all antibiotic prescriptions)	76	86	90	93	97
Repeat prescriptions (% of all antibiotic prescriptions)	3	7	10	14	24
Coded indication (% of all antibiotic prescriptions)	10	48	58	65	75
Respiratory (% of all antibiotic prescriptions)	6	31	36	42	52
Genito-urinary (% of all antibiotic prescriptions)	1	7	8	11	16
Skin (% of all antibiotic prescriptions)	2	8	10	12	16
Other specific (% of all antibiotic prescriptions)	0	1	1	2	3
Non-coded indications (% of all antibiotic prescriptions)	24	35	42	51	90
No codes recorded (% of all antibiotic prescriptions)	1	3	6	11	28
Non-specific codes recorded (% of all antibiotic prescriptions)	12	19	24	29	59

Column percents are not expected to sum to 100 as different family practices may be represented for the same centile in different rows

For peer review only

<u>Legends for Figures</u>

Figure 1: Age- and sex-standardised antibiotic prescribing rates per 1,000 patient years for coded and not coded indications from 2002 to 2017.

Figure 2: Age-standardised rates of serious bacterial infections per 1,000 patient years from 2002 to 2017. Red lines, female; blue lines, male; shaded areas, 95% confidence intervals.

Figure 3: Forest plot showing the adjusted rate ratio for each type of serious bacterial infection for 20% higher total antibiotic prescribing (red) or 20% higher proportion of antibiotic prescriptions with specific coded indications recorded (grey). Estimates were adjusted for each variable shown and gender, age-group, comorbidity, deprivation fifth, region and year (including quadratic and cubic terms).

For peer review only

For peer review only - http://bmjopen.bmj.com/site/about/guidelines.xhtml

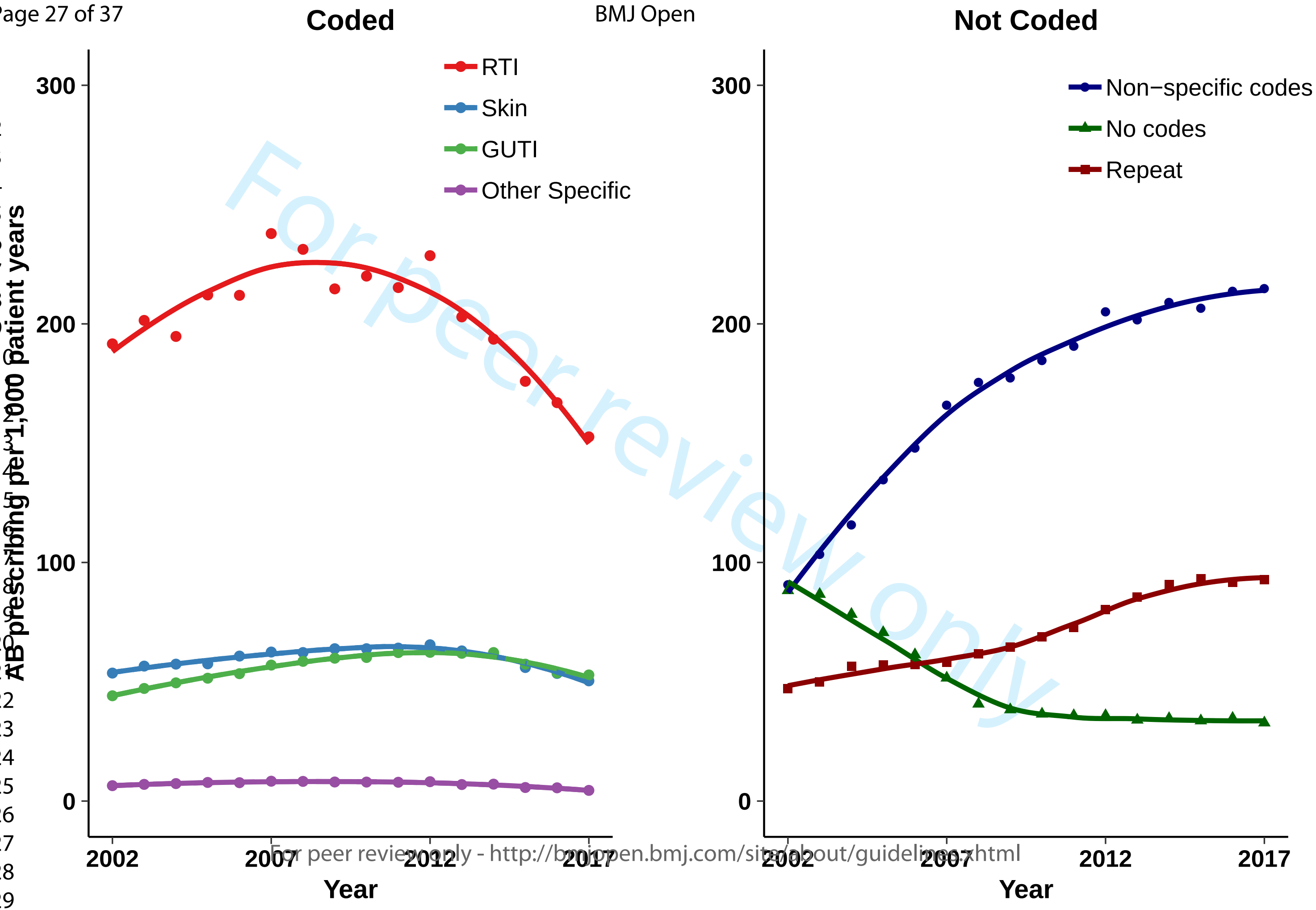

For peer review only

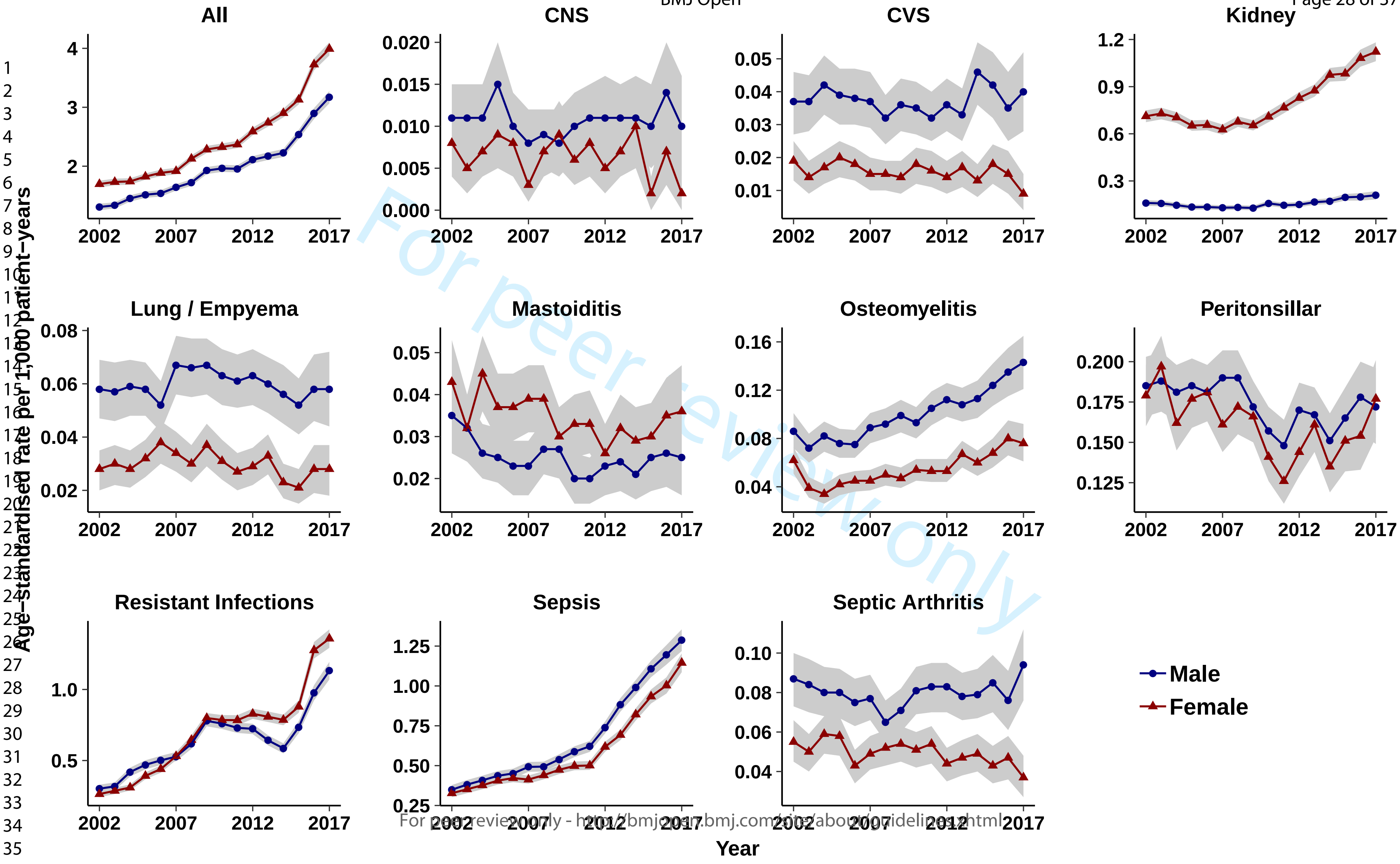

For peer review only

For peer review only - http://bmjopen.bmj.com/site/about/guidelines.xhtml

	All AB Prescribing				Medical Coding			
**Outcome**	**RR**	**LL**	**UL**	**P value**	**RR**	**LL**	**UL**	**P value**
CNS infection	1.01	0.93	1.10	0.853	1.14	1.01	1.30	0.039
CVS infection	0.96	0.87	1.06	0.402	1.22	1.06	1.41	0.005
Kidney infection	1.00	0.96	1.04	0.921	1.28	1.20	1.35	<0.001
Lung abscess / Empyema	0.94	0.88	1.00	0.038	1.15	1.05	1.25	0.002
Mastoiditis	1.08	0.97	1.20	0.179	1.52	1.29	1.79	<0.001
Osteomyelitis	0.97	0.93	1.02	0.287	1.14	1.06	1.23	0.001
Peritonsillar abscess	0.98	0.94	1.02	0.334	1.21	1.14	1.28	<0.001
Resistant infection	1.04	0.98	1.11	0.161	1.38	1.26	1.51	<0.001
Sepsis	1.03	0.99	1.08	0.185	1.23	1.15	1.31	<0.001
Septic Arthritis	0.96	0.91	1.01	0.123	1.23	1.14	1.33	<0.001
**Combined**	**1.03**	**1.00**	**1.06**	**0.074**	**1.24**	**1.18**	**1.29**	**<0.001**

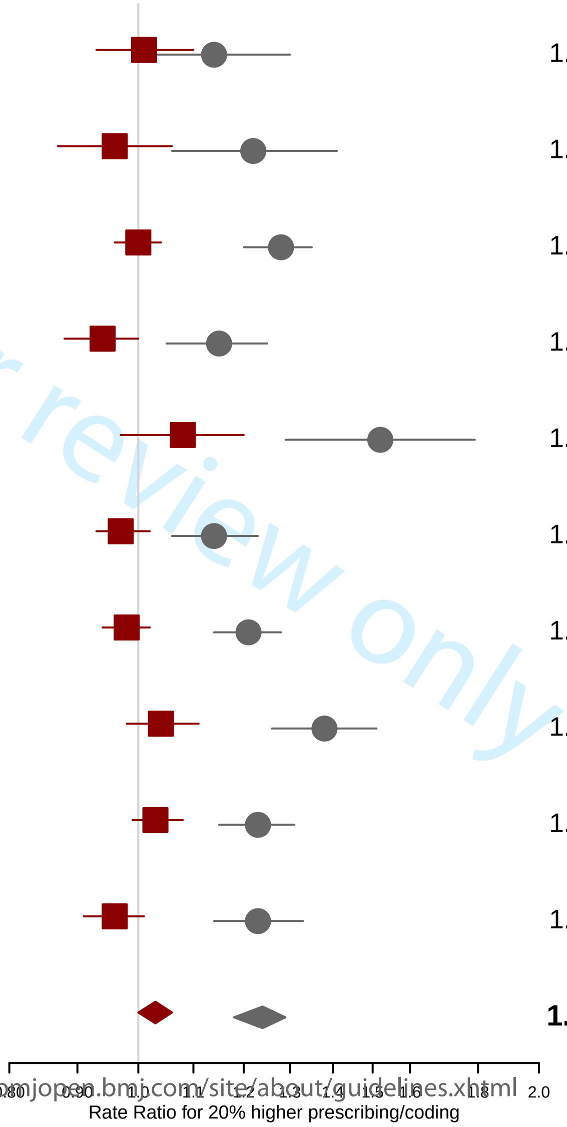

For peer review only

Supplementary Table 1: Outline of statistical models employed for analysis. (Please see text for further explanation).

**Model 1**	Antibiotic_count ~ year+year^2+year^3+age_group+gender+comorbidity+deprivation+region+offset=log(person-years)+random_intercept(family_practice), model=Poisson
**Model 2**	Serious_bacterial_infection_count ~ Family_Practice_AB_Prescribing_Estimate+Family_Practice_Consultations_Coded_Estimate+year+year^2+year^3+gender+ age_group+deprivation+region+offset=log(person-years)+random_intercept(family_practice), model=Poisson

For peer review only

Gulliford et al. Supplementary Data

Figure 1: Flowchart showing classification of antibiotic prescriptions 2002 to 2017. Figures are frequencies (percent of total number of antibiotic prescriptions).

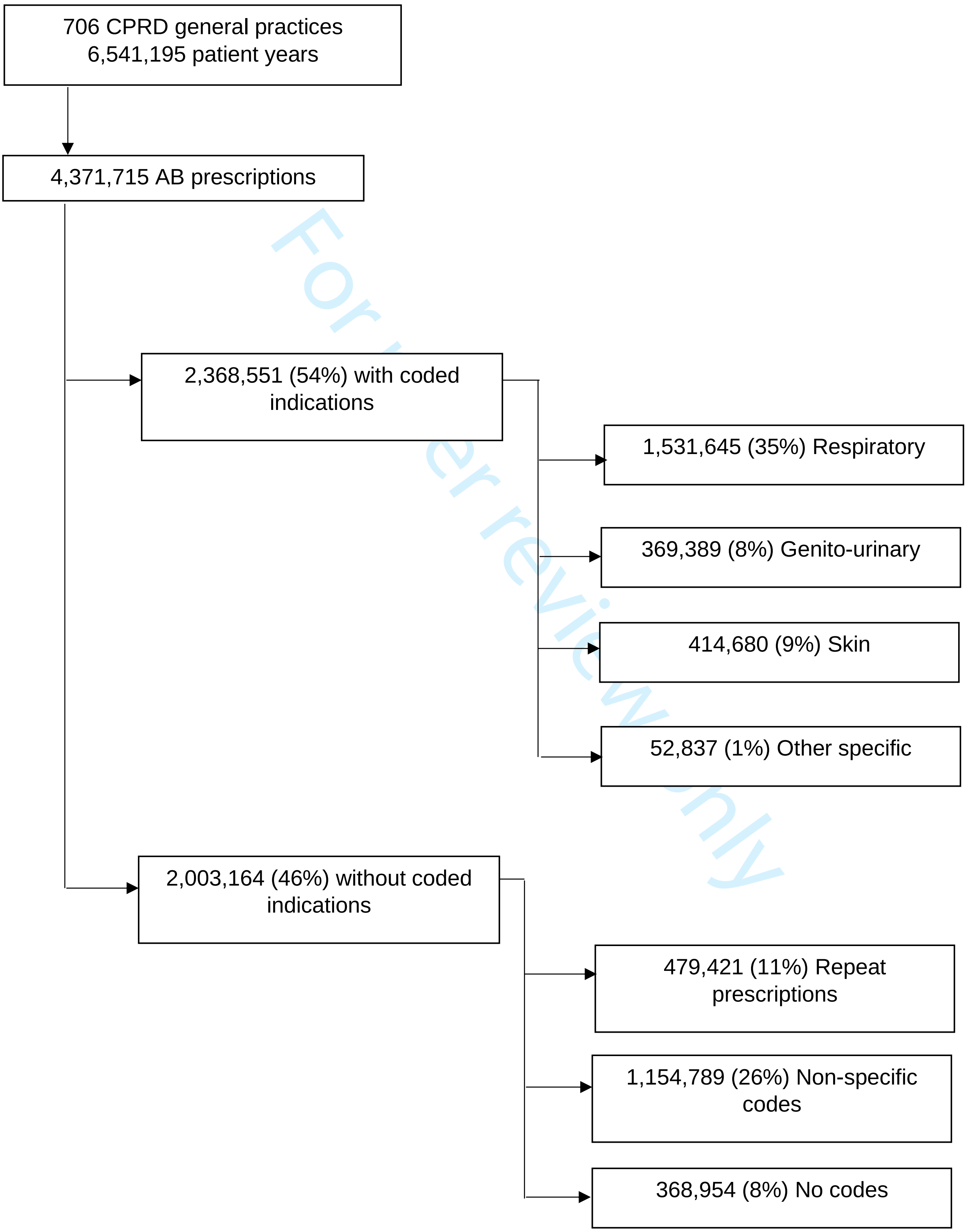

For peer review only - http://bmjopen.bmj.com/site/about/guidelines.xhtml

Gulliford et al. Supplementary Data

Supplementary Table 2: Characteristics of sample and antibiotic prescriptions by time period. Figures are frequencies (column percent).

	2002 to 2006	2007 to 2012	2013 to 2017
**Number of general practices**	652	672	589
**Number of patients contributing person time[a]**	548,558	576,985	439,627
**Number of person years**	2,253,436	2,768,176	1,519,582
Age 0 to 4	275,539	313,806	104,688
Age 5 to 14	371,352	611,610	393,224
Age 85+	169,709	216,966	111,606
Comorbidity present[a]	835,565	1,147,828	686,777
**Number of antibiotic prescriptions**	1,422,009	1,941,102	1,008,604
Acute AB prescriptions	1,289,615 (91)	1,739,666 (90)	863,013 (86)
for RTI	534,535 (38)	705,262 (36)	291,848 (29)
for GUTI	115,928 (8)	166,336 (9)	87,125 (9)
for skin infection	137,936 (10)	184,420 (10)	92,324 (9)
Other specific codes recorded	18,277 (1)	24,849 (1)	9,711 (1)
Non-specific codes recorded	290,472 (20)	537,110 (28)	327,207 (32)
No codes recorded	192,467 (14)	121,689 (6)	54,798 (5)
Repeat AB prescriptions	132,394 (9)	201,436 (10)	145,591 (15)

[a] figures were rounded to nearest whole number; [b]based on seasonal-influenza risk status

For peer review only

Gulliford et al. Supplementary Data

Supplementary Figure 2: Age-standardised antibiotic prescribing rates per 1,000 patient years for males and females from 2002 to 2017.

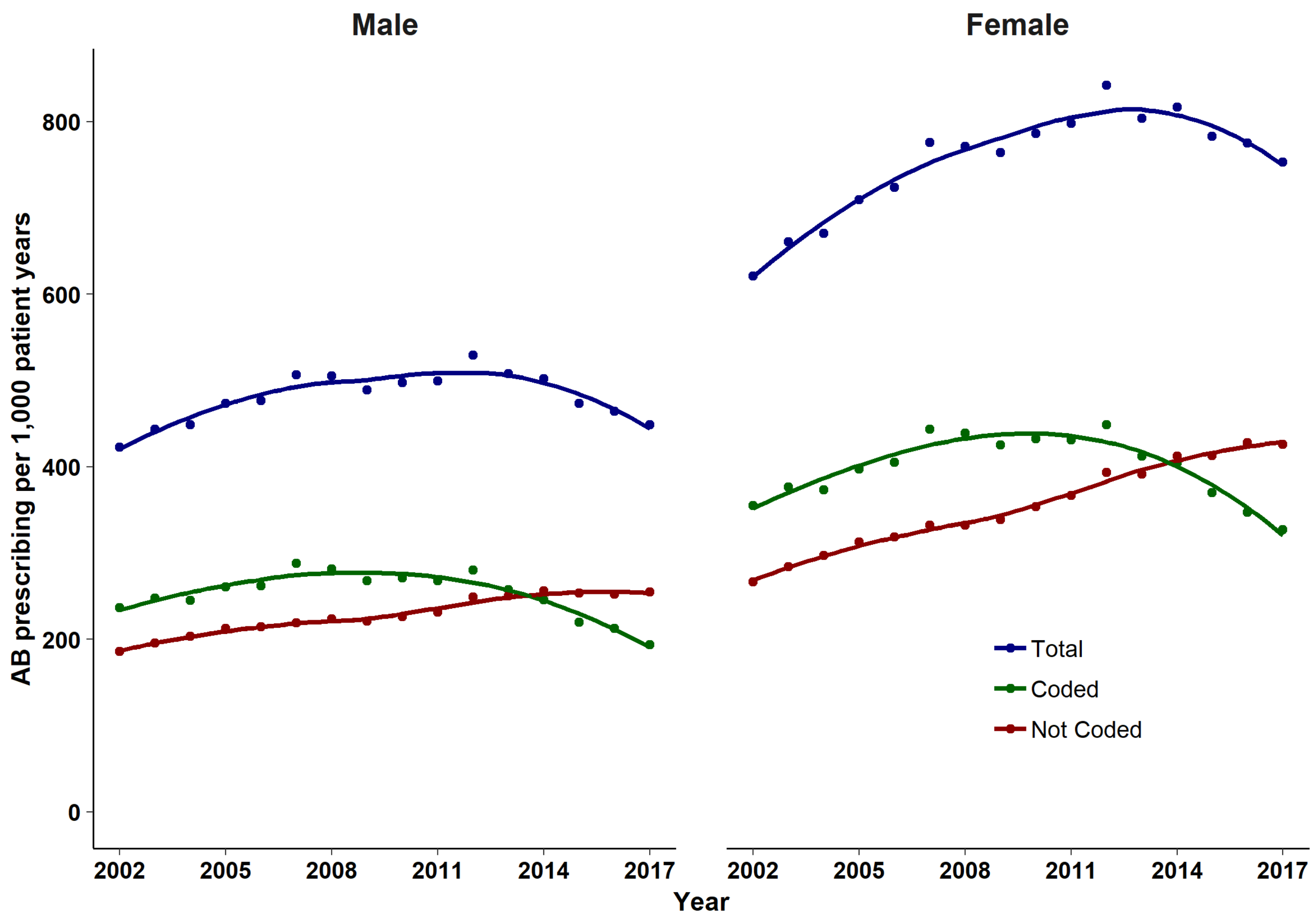

For peer review only - http://bmjopen.bmj.com/site/about/guidelines.xhtml

Gulliford et al. Supplementary Data

Supplementary Table 3: Distribution of serious bacterial infection events 2002 to 2017 by age-group and gender. Figures are frequencies.

	CNS infection	CVS infection	Kidney infection	Lung abscess / empyema	Mastoiditis	Osteomyelitis	Peritonsillar abscess	AB resistant infections	Sepsis	Septic arthritis
All	576	1,697	30,827	2,932	1,970	4,921	11,338	42,185	39,059	4,254
Male	352	1,144	4,997	1,903	814	3,055	6,021	18,312	18,999	2,496
Female	224	553	25,830	1,029	1,156	1,866	5,317	23,873	20,060	1,758
**Age-group (years)**										
0 to 4	11	20	198	138	178	138	73	576	469	147
5 to 9	17	18	386	106	153	118	232	409	334	104
10 to 14	17	17	474	60	111	167	465	308	244	93
15 to 24	47	42	6,140	106	167	152	3,428	1,528	970	129
25 to 34	38	92	5,523	149	203	160	2,621	2,444	1,474	243
35 to 44	65	146	5,176	294	280	392	2,483	3,089	2,164	392
45 to 54	115	189	4,519	438	270	635	1,079	4,001	3,345	555
55 to 64	105	274	3,725	561	255	865	553	5,045	5,385	678
65 to 74	90	407	2,562	525	210	937	285	8,252	7,817	775
75 to 84	58	365	1,548	423	109	924	94	9,469	9,646	727
85+	13	127	576	132	34	433	24	7,064	7,211	411

For peer review only

For peer review only - http://bmjopen.bmj.com/site/about/guidelines.xhtml

Gulliford et al. Supplementary Data

Supplementary Table 4: Age- and sex-standardised incidence rates per 1,000 patient-years for serious bacterial infections by quartile of antibiotic prescribing.

Group	General practice antibiotic prescribing[a]		General practice medical coding[a]	
	Lowest fourth of general practices	Highest fourth of general practices	Lowest fourth of general practices	Highest fourth of general practices
All	1.86 (1.83 to 1.88)	2.23 (2.20 to 2.25)	1.94 (1.91 to 1.96)	2.39 (2.37 to 2.42)
CNS Infection	0.008 (0.007 to 0.010)	0.009 (0.008 to 0.011)	0.008 (0.007 to 0.009)	0.010 (0.009 to 0.012)
CVS infection	0.024 (0.021 to 0.027)	0.026 (0.023 to 0.028)	0.026 (0.024 to 0.029)	0.027 (0.025 to 0.030)
Kidney Infection	0.40 (0.39 to 0.41)	0.49 (0.48 to 0.50)	0.37 (0.37 to 0.38)	0.55 (0.53 to 0.56)
Lung abscess / empyema	0.042 (0.039 to 0.045)	0.045 (0.042 to 0.049)	0.044 (0.041 to 0.047)	0.049 (0.046 to 0.053)
Mastoiditis	0.025 (0.022 to 0.027)	0.033 (0.030 to 0.036)	0.021 (0.019 to 0.023)	0.036 (0.033 to 0.039)
Osteomyelitis	0.071 (0.067 to 0.075)	0.073 (0.069 to 0.077)	0.071 (0.067 to 0.075)	0.081 (0.077 to 0.086)
Peritonsillar abscess	0.16 (0.15 to 0.17)	0.16 (0.16 to 0.17)	0.14 (0.14 to 0.15)	0.17 (0.17 to 0.18)
Resistant infections & C. difficile	0.50 (0.49 to 0.51)	0.68 (0.67 to 0.69)	0.63 (0.62 to 0.64)	0.73 (0.72 to 0.74)
Sepsis	0.57 (0.56 to 0.58)	0.65 (0.63 to 0.66)	0.56 (0.55 to 0.57)	0.67 (0.66 to 0.68)
Septic arthritis	0.064 (0.059 to 0.068)	0.064 (0.060 to 0.068)	0.057 (0.053 to 0.061)	0.068 (0.064 to 0.072)

[a]quartiles were estimated from a hierarchical regression model adjusting for age-group, gender, comorbidity, region, deprivation and year

For peer review only - http://bmjopen.bmj.com/site/about/guidelines.xhtml

Gulliford et al. Supplementary Data

Supplementary Figure 3: Forest plot showing the adjusted rate ratio for each type of serious bacterial infection for 20% higher total AB prescribing (red) or repeat AB prescribing (grey). Estimates were adjusted for each variable shown and gender, age-group, comorbidity, deprivation fifth, region, year (including quadratic and cubic terms).

■ Acute AB prescribing ● Repeat AB prescribing

	Acute Prescribing				Repeat Prescribing			
**Outcome**	**RR**	**LL**	**UL**	**P value**	**RR**	**LL**	**UL**	**P value**
CNS infection	1.01	0.93	1.09	0.812	0.99	0.96	1.02	0.593
CVS infection	0.94	0.86	1.03	0.186	1.02	0.99	1.06	0.225
Kidney infection	0.98	0.94	1.01	0.197	1.03	1.02	1.05	<0.001
Lung abscess / Empyema	0.92	0.87	0.97	0.003	1.03	1.00	1.05	0.025
Mastoiditis	1.04	0.94	1.15	0.417	1.04	1.00	1.09	0.051
Osteomyelitis	0.96	0.91	1.00	0.055	1.03	1.01	1.05	0.008
Peritonsillar abscess	0.96	0.92	0.99	0.022	1.03	1.02	1.05	<0.001
Resistant infection	1.04	0.98	1.10	0.203	1.00	0.98	1.03	0.752
Sepsis	1.03	0.99	1.07	0.186	1.00	0.98	1.02	0.972
Septic Arthritis	0.94	0.89	0.98	0.007	1.03	1.01	1.05	0.006
**Combined**	**1.02**	**0.99**	**1.05**	**0.227**	**1.01**	**1.00**	**1.02**	**0.054**

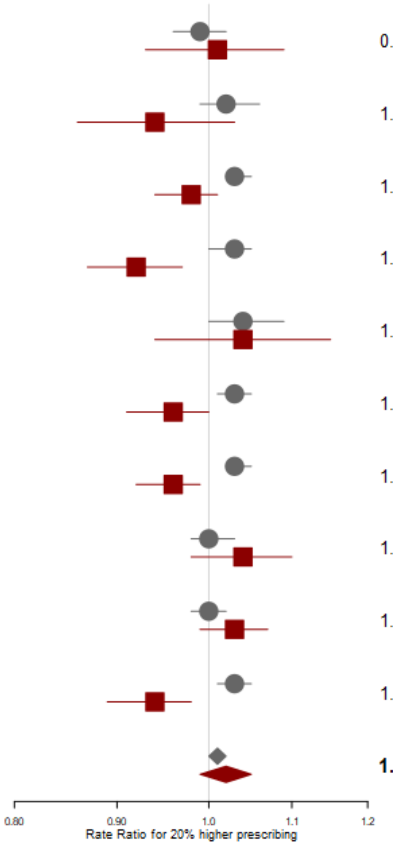

STROBE Statement—Checklist of items that should be included in reports of ***cohort studies***

	Item No	Recommendation	
**Title and abstract**	1	(*a*) Indicate the study's design with a commonly used term in the title or the abstract	P1
		(*b*) Provide in the abstract an informative and balanced summary of what was done and what was found	P3
**Introduction**			
Background/rationale	2	Explain the scientific background and rationale for the investigation being reported	P4-5
Objectives	3	State specific objectives, including any prespecified hypotheses	P5
**Methods**			
Study design	4	Present key elements of study design early in the paper	P6
Setting	5	Describe the setting, locations, and relevant dates, including periods of recruitment, exposure, follow-up, and data collection	P6
Participants	6	(*a*) Give the eligibility criteria, and the sources and methods of selection of participants. Describe methods of follow-up	P6
		(*b*) For matched studies, give matching criteria and number of exposed and unexposed	N/A
Variables	7	Clearly define all outcomes, exposures, predictors, potential confounders, and effect modifiers. Give diagnostic criteria, if applicable	P6-8
Data sources/ measurement	8*	For each variable of interest, give sources of data and details of methods of assessment (measurement). Describe comparability of assessment methods if there is more than one group	P6
Bias	9	Describe any efforts to address potential sources of bias	P8/9
Study size	10	Explain how the study size was arrived at	P6
Quantitative variables	11	Explain how quantitative variables were handled in the analyses. If applicable, describe which groupings were chosen and why	P6-8
Statistical methods	12	(*a*) Describe all statistical methods, including those used to control for confounding	P8/9
		(*b*) Describe any methods used to examine subgroups and interactions	P8/9
		(*c*) Explain how missing data were addressed	P8/9
		(*d*) If applicable, explain how loss to follow-up was addressed	N/A
		(*e*) Describe any sensitivity analyses	P12/13
**Results**			
Participants	13*	(a) Report numbers of individuals at each stage of study—eg numbers potentially eligible, examined for eligibility, confirmed eligible, included in the study, completing follow-up, and analysed	P10, ST2
		(b) Give reasons for non-participation at each stage	N/A
		(c) Consider use of a flow diagram	SF1
Descriptive data	14*	(a) Give characteristics of study participants (eg demographic, clinical, social) and information on exposures and potential confounders	ST2
		(b) Indicate number of participants with missing data for each variable of interest	SF1
		(c) Summarise follow-up time (eg, average and total amount)	P8, P10
Outcome data	15*	Report numbers of outcome events or summary measures over time	ST3
Main results	16	(*a*) Give unadjusted estimates and, if applicable, confounder-adjusted	Fig3

For peer review only - http://bmjopen.bmj.com/site/about/guidelines.xhtml

		estimates and their precision (eg, 95% confidence interval). Make clear which confounders were adjusted for and why they were included	P8/9, ST1
		(*b*) Report category boundaries when continuous variables were categorized	P7/8
		(*c*) If relevant, consider translating estimates of relative risk into absolute risk for a meaningful time period	P13
Other analyses	17	Report other analyses done—eg analyses of subgroups and interactions, and sensitivity analyses	P11
**Discussion**			
Key results	18	Summarise key results with reference to study objectives	P13
Limitations	19	Discuss limitations of the study, taking into account sources of potential bias or imprecision. Discuss both direction and magnitude of any potential bias	P14-15
Interpretation	20	Give a cautious overall interpretation of results considering objectives, limitations, multiplicity of analyses, results from similar studies, and other relevant evidence	P16
Generalisability	21	Discuss the generalisability (external validity) of the study results	P16
**Other information**			
Funding	22	Give the source of funding and the role of the funders for the present study and, if applicable, for the original study on which the present article is based	P17

*Give information separately for exposed and unexposed groups.

Note: An Explanation and Elaboration article discusses each checklist item and gives methodological background and published examples of transparent reporting. The STROBE checklist is best used in conjunction with this article (freely available on the Web sites of PLoS Medicine at http://www.plosmedicine.org/, Annals of Internal Medicine at http://www.annals.org/, and Epidemiology at http://www.epidem.com/). Information on the STROBE Initiative is available at http://www.strobe-statement.org.

For peer review only
